# Effective coverage of facility delivery in Bangladesh, Haiti, Malawi, Nepal, Senegal, and Tanzania

**DOI:** 10.1371/journal.pone.0217853

**Published:** 2019-06-11

**Authors:** Wenjuan Wang, Lindsay Mallick, Courtney Allen, Thomas Pullum

**Affiliations:** 1 The Demographic and Health Surveys (DHS) Program, Division of International Health and Development, ICF, Rockville, Maryland, United States of America; 2 Avenir Health, Glastonbury, Connecticut, United States of America; BRAC, BANGLADESH

## Abstract

**Background:**

The persistence of preventable maternal and newborn deaths highlights the importance of quality of care as an essential element in coverage interventions. Moving beyond the conventional measurement of crude coverage, we estimated effective coverage of facility delivery by adjusting for facility preparedness to provide delivery services in Bangladesh, Haiti, Malawi, Nepal, Senegal, and Tanzania.

**Methods:**

The study uses data from Demographic and Health Surveys (DHS) and Service Provision Assessments (SPA) in Bangladesh (2014 DHS and 2014 SPA), Haiti (2012 DHS and 2013 SPA), Malawi (2015–16 DHS and 2013–14 SPA), Nepal (2016 DHS and 2015 SPA), Senegal (2016 DHS and 2015 SPA), and Tanzania (2015–16 DHS and 2014–15 SPA). We defined effective coverage as the mathematical product of crude coverage and quality of care. The coverage of facility delivery was measured with DHS data and quality of care was measured with facility data from SPA. We estimated effective coverage at both the regional and the national level and accounted for type of facility where delivery care was sought.

**Findings:**

The findings from the six countries indicate the effective coverage ranges from 24% in Haiti to 66% in Malawi, representing substantial reductions (20% to 39%) from crude coverage rates. Although Malawi has achieved almost universal coverage of facility delivery (93%), effective coverage was only 66%.vSuch gaps between the crude coverage and the effective coverage suggest that women delivered in health facility but did not necessarily receive an adequate quality of care. In all countries except Malawi, effective coverage differed substantially among the country’s regions of the country, primarily due to regional variability in coverage.

**Interpretation:**

Our findings reinforce the importance of quality of obstetric and newborn care to achieve further reduction of maternal and newborn mortality. Continued efforts are needed to increase the use of facility delivery service in countries or regions where coverage remains low.

## Introduction

Despite global increases in coverage of facility delivery, the reduction in maternal and neonatal deaths remains limited.[1] Crude coverage describes the use of care services, but does not provide information about the quality of care received. Examining the quality of maternal and newborn healthcare services centers upon the idea that skilled care provided at delivery, supported by well-equipped facilities, is critical for identifying and addressing complications in time for women and newborns to receive treatment and to save lives.[2] Moving beyond the conventional measurement of crude coverage, effective coverage combines both use and quality into one measurement, which can be understood as the fraction of the maximum health gain actually delivered through the health system to the population in need.[3, 4]

The concept of effective coverage first appeared several decades ago. The Tanahashi framework illustrated effective coverage as the final stage of service provision after availability of health services, physical accessibility to services, acceptability by those in need, and actual use of the service.[5] Shengelia et al. proposed a framework for effective coverage that integrates need, use, and quality [4]. Research on effective coverage has resumed recently but remained limited.[6] The few studies of effective coverage of maternal and child health services produce striking reductions of crude coverage. In Ghana, linking facility data to population data by districts, two-thirds of all births occurred in a health facility, but only one in every four births occurred in a high-quality facility.[7] Similarly, in Tanzania, using a high quality standard that facilities have 90% of required items, the estimate of effective coverage reduced crude coverage from 80% to zero.[8] In a study of 17 countries, using a stringent quality measurement cut-off of 20 or more out of 23 essential items, median coverage of facility delivery fell from 42% to 28%.[9]

When estimating effective coverage, measuring the quality of care can be challenging.[10] Of concern for quality of delivery care, there is no single set of standard measures used to assess quality.[1, 7, 11–13] Donabedian defined quality by components of structure, process, and outcome [14], which describe the health facility setting, care delivered to the client, and the client outcome. Many studies of quality of care in facility delivery focus on structural inputs.[7, 8, 13, 15, 16] Assessments of care practices provided has been limited,[11] and such observations are time-consuming, prone to measurement error, and subject to their own quality limitations, particularly in resource-constrained settings.[11, 17] Thus, service readiness assessments such as the World Health Organization (WHO) Service Availability and Readiness Assessment (SARA),[18] and The Demographic and Health Survey Program’s Service Provision Assessment (SPA), have been used as substitutes. These tools provide an overview of facility structural capacity to provide services but do not routinely include observation of actual service delivery.

The primary objective of this study is to estimate the effective coverage of obstetric and newborn care with a refined approach. This method takes into account different types of facilities where women delivered their births. We also estimated the uncertainty of the effective coverage estimates, which has not been commonly done in previous research on effective coverage. In measuring quality of care, we use a wide range of input-based quality of care indicators to provide a comprehensive assessment of the readiness of facilities to deliver obstetric and newborn care in these We link data from nationally representative household surveys -Demographic and Health Surveys (DHS)- with data from surveys of health facilities–SPA surveys in six countries. These countries were selected primarily because of the availability of closely timed DHS survey and SPA that can be linked. All six countries have high maternal and newborn mortality,[19, 20] which makes this analysis particularly relevant for these countries.

## Data and methods

### Data

This analysis is based on data from DHS and SPA surveys in six countries: Bangladesh, Haiti, Malawi, Nepal, Senegal, and Tanzania. All countries had recent DHS and SPA surveys completed within two years of each other. Both DHS and SPA surveys are primarily funded by the United States Agency for International Development and carried out by an in-country implementing agency (usually the country’s ministry of health or statistics office) with technical assistance from The DHS Program.

The DHS surveys are population-based household surveys that provides representative data on health indicators at national and regional levels. All women age 15–49 in selected households with a birth in the five years before the survey are interviewed about delivery care, including place of delivery for all live births during this period. This study focuses on delivery care received for live births in just the two years preceding the survey, to better synchronize the timing of the DHS and SPA data.

The SPA is a health facility-based survey designed to provide information on the availability and quality of preventive and curative health services. In each country except Haiti and Malawi, where the SPA was a facility census, a sample of formal health facilities was selected to represent the country and the administrative regions, by type of facility and by managing authority. This study focuses on facilities that provide delivery services, using data from the facility inventory and provider interviews. Table 1 provides the number of births and health facilities included in the analysis.

**Table 1:** Description of SPA and DHS samples included in the study.

Country	DHS survey year	Number of births in the two years preceding the survey	SPA survey year	Number of facilities with delivery services					
				Non-CEmOC facilities		CEmOC facilities		All facilities	
				Unweighted	Weighted	Unweighted	Weighted	Unweighted	Weighted
Bangladesh	2014	3,147	2014	520	267	66	13	586	280
Haiti	2012	2,747	2013	379	379	10	10	389	389
Malawi	2015–16	6,596	2013–14	529	517	11	11	540	528
Nepal	2016	1,978	2015	585	448	36	9	621	457
Senegal	2016	2,311	2015	358	361	4	2	364	363
Tanzania	2015–16	4,327	2014–15	905	896	46	8	951	905

### Statistical analysis

Effective coverage is calculated among individuals in need of care as the mathematical product of the use of the service and the quality of care provided.[4, 21] We first calculate the two components—coverage of facility delivery and the quality of facility delivery services.

#### Coverage of facility delivery

We estimated the coverage of facility delivery based on DHS data as the percentage of births in the two years preceding the survey that were delivered in a health facility. We disaggregated the coverage by type of facility where the delivery occurred, because women may seek delivery care in a range of facilities with varied preparedness. For each of the six countries, facility types were harmonized between the DHS and SPA. S1 Tables provides a summary of the harmonized classifications for each country.

#### Facility readiness to provide delivery care

This study focused on the structural aspect of quality of care, which refers to the physical attributes of a health facility including infrastructure, equipment, supplies, commodities, and the availability of trained personnel; in other words, service or facility readiness. We measured facility readiness with a composite score computed with a set of readiness indicators of obstetric and newborn care. Indicator selection was guided by three references: the World Health Organization (WHO) SARA Manual,[18] the indicators suggested by the Newborn Indicator Technical Working Group,[22] and a comprehensive systematic review by Gabrysch et al.[15] S1 Tables provides definitions of these indicators.

We calculated the composite readiness score using an equal-weight approach. This has proved to be the preferable method to create a composite measurement compared with other weighting schemes.[23, 24]Equal weight was given to six domains of readiness and to all indicators within the same domain; the sum of all domains was standardized to have a maximum of 100. Since non-CEmOC facilities are not expected to provide C-sections and safe blood transfusion, these two indicators were included in the calculation of readiness scores only for CEmOC facilities. Given this standardization, a facility’s score is interpreted as the percentage of the highest possible readiness that the facility could have.

#### Estimating effective coverage

Effective coverage was estimated at both the regional and the national level, accounting for types of facilities where delivery care was sought.[25] The national estimates are improved by taking regional variations into account because regions differ in the use of each type of facility and in readiness among facilities in the same category. In most countries, the regions are administrative regions or provinces for which both DHS data and SPA data are representative. In Tanzania, regions were further grouped into nine geographic zones to allow for a large sample size in each zone, therefore reduced sampling errors.

At the regional level, the effective coverage is the summation of effective coverage of each type of facility that is constructed as the product of the coverage and readiness estimates:

$${EC}_{r}=\sum_{j}{(C}_{rj}*Q_{rj})$$

where *EC*_*r*_ represents effective coverage in region *r*,

*C*_*rj*_ is the proportion of births delivered in facility type *j* in region *r*,

and

*Q*_*rj*_ is the average readiness score of facility type *j* in region *r*.

We accounted for the DHS sampling weight when estimating facility delivery coverage and the SPA sampling weight when calculating readiness scores. The calculated readiness score for a specific facility category is an average score of all facilities in the same category.

The national effective coverage is the summation of regional effective coverage weighted by the proportion of births in each region:

$${EC}_{T}=\sum_{r}{EC}_{r}*w_{r}$$

Where *EC*_*T*_ represents the national level effective coverage and *w*_*r*_ represents the proportion of births in region *r*.

The uncertainty of the estimates of effective coverage was assessed with an approximation procedure referred to as the “delta” method.[26] A detailed description of variance estimation of effective coverage is provided in S1 File.

## Results

Analyzing the distribution of facilities by type, we found that government health facilities are the major providers of delivery care in all six countries (Fig A in S1 Figs). About 80% or more of facilities offering delivery care in Bangladesh, Tanzania, Nepal, and Senegal are government health facilities. Private facilities, especially private not-for-profit facilities, represent a larger share in Haiti and Malawi than in other countries. Most of the countries rely on lower-level facilities such as health posts or sub-health posts and dispensaries for delivery care. Government hospitals have a small share ranging from 1% in Senegal to 11% in Haiti.

We examined the availability of tracer items that are important for providing delivery care in each country at the regional level (only for non-CEmOC facilities) and at the national level (Tables C-H in S1 Tables). We found that facilities, especially non-CEmOC facilities, often lack equipment, medicines, supplies, or trained personnel needed to provide high quality of care. In all countries except Malawi, less than a third of non-CEmOC facilities had a 24/7 skilled birth attendant. Many non-CEmOC facilities lacked a functional emergency transportation system, available in only 30% of facilities in Bangladesh and Haiti Equipment such as a manual vacuum extractor, vacuum aspirator kit, were seldom observed, with either supply available in less than 10% of facilities in Tanzania, less than 20% in Haiti or Nepal, and less than 30% in Bangladesh,. In Bangladesh, availability of medicines and commodities ranged from 17% for hydrocortisone to 32% with an injectable uterotonic, and 33% with IV solution with an infusion set. Among non-CEmOC facilities, the regions often had similar items available at their facilities. While health facilities performed well in providing immediate newborn care services, provision of basic emergency obstetric care and newborn resuscitation was limited, a finding that was consistent across regions.

As expected, the availability of items was higher among CEmOC facilities.In all the countries, for both CEmOC and non-CEmOC facilities, the domain with the most limited availability was guidelines, training, and supervision. Less than one third of CEmOC facilities had a provider trained in CEmOC in 4 countries- Nepal (11%), Senegal (12%), Bangladesh (29%), and Tanzania (31%).

Readiness scores were calculated by facility type and region. We present the results with the corresponding coverage of facility delivery to facilitate the comparisons between readiness and use (Figs B-Min S1 Figs). In all countries, hospitals, whether private or public, were typically the type of facility most ready to provide delivery care, whereas lower-level facilities were much less prepared. For example, in Bangladesh, public hospitals had the highest readiness score, with 77% of the maximum capacity to provide delivery care services, but public union facilities had a readiness score of only 37%. Despite the poor readiness of these lower-level facilities, many were reported by women as one of the major sources of delivery care. In fact, in several countries the type of facility least ready to provide delivery care was the most commonly reported place of delivery. In Nepal, for example, despite having the lowest readiness score, government health posts were widely used in Provinces 6 and 7. In Tanzania, government dispensaries had the lowest readiness score but were commonly reported as a source for delivery care., the facility type with the lowest readiness score. A similar pattern was found in Senegal: the most commonly used type of facility, the government health post, had low service readiness scores compared with government hospitals and health centers.

**Fig 1:**
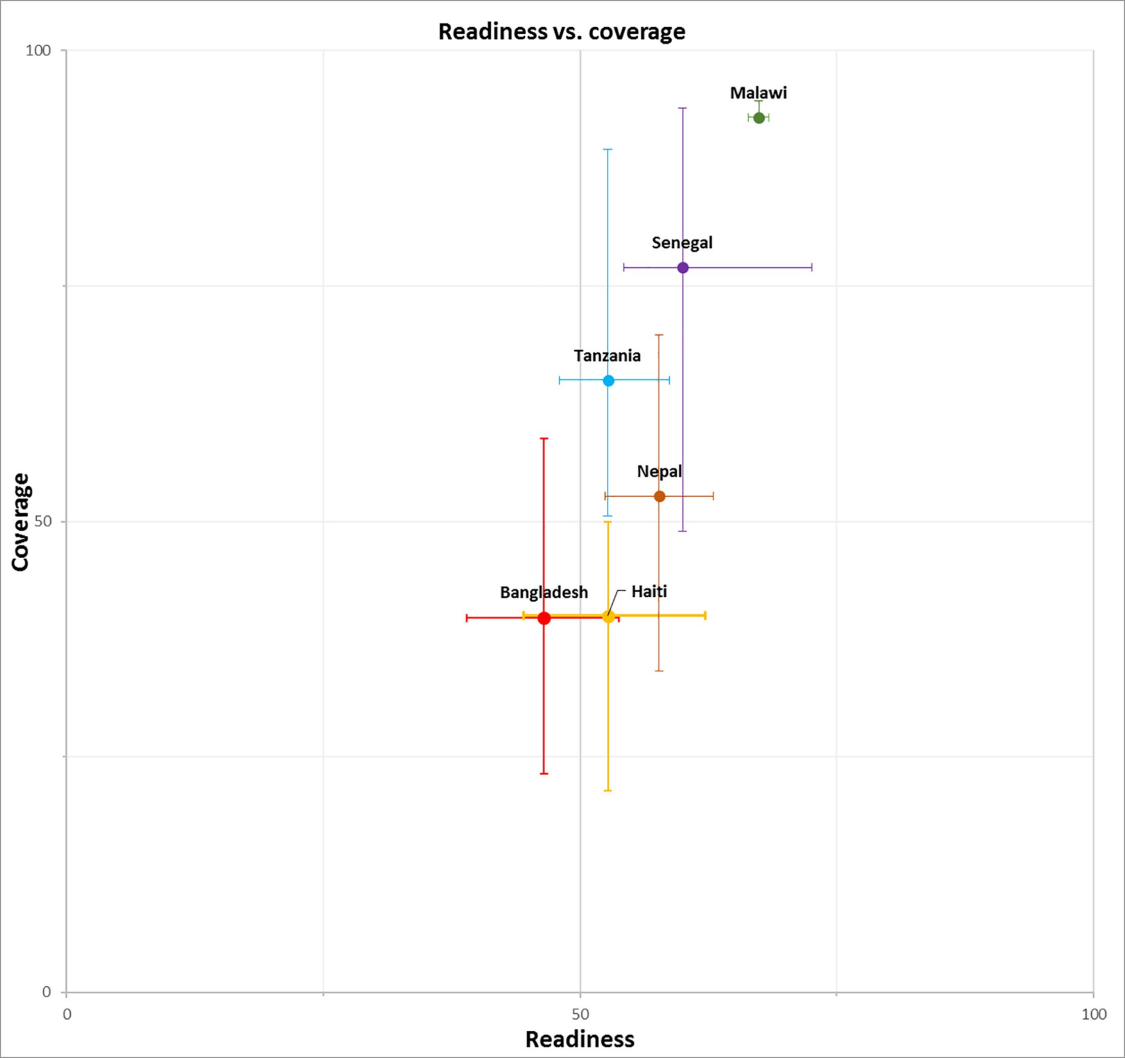
National readiness score versus coverage and regional variations.

Fig 1 depicts the countries’ national coverage of facility delivery against their facilities’ readiness score, as well as the range among their regions. For each country, the horizontal whisker indicates the range of the readiness score among the country’s regions, and the vertical whisker represents the range of the facility delivery coverage among the regions. The longer the whisker, the greater the variability among the regions. In four countries—Malawi, Senegal, Tanzania, and Nepal—national averages of coverage and readiness fell in quadrant I, indicating that both the national coverage and the readiness score were higher than 50%. Malawi had the highest coverage of facility delivery and the greatest readiness. Bangladesh had the lowest coverage and readiness, both lower than 50%. Haiti is in quadrant IV, with a readiness score above 50% but coverage below 50%. All countries demonstrated a larger regional variability in coverage than in readiness except for Malawi, where the regions had similar levels of coverage and readiness. Senegal had the greatest range of crude coverage of both facility delivery and facility readiness by region. Fig N in S1 Figs presents regional levels of readiness against coverage for each country.

**Fig 2:**
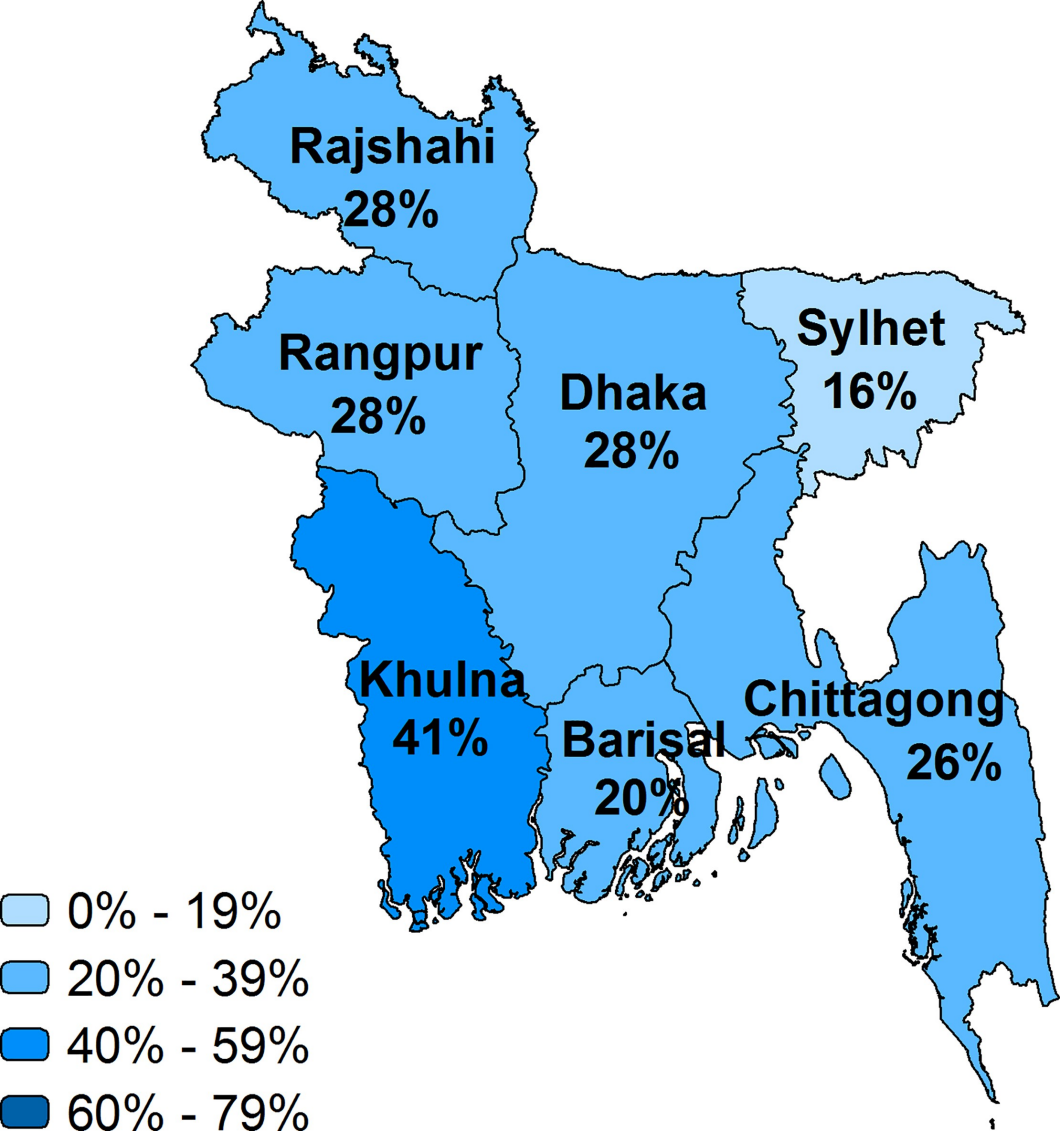
Effective coverage of facility delivery by region, Bangladesh.

**Fig 3:**
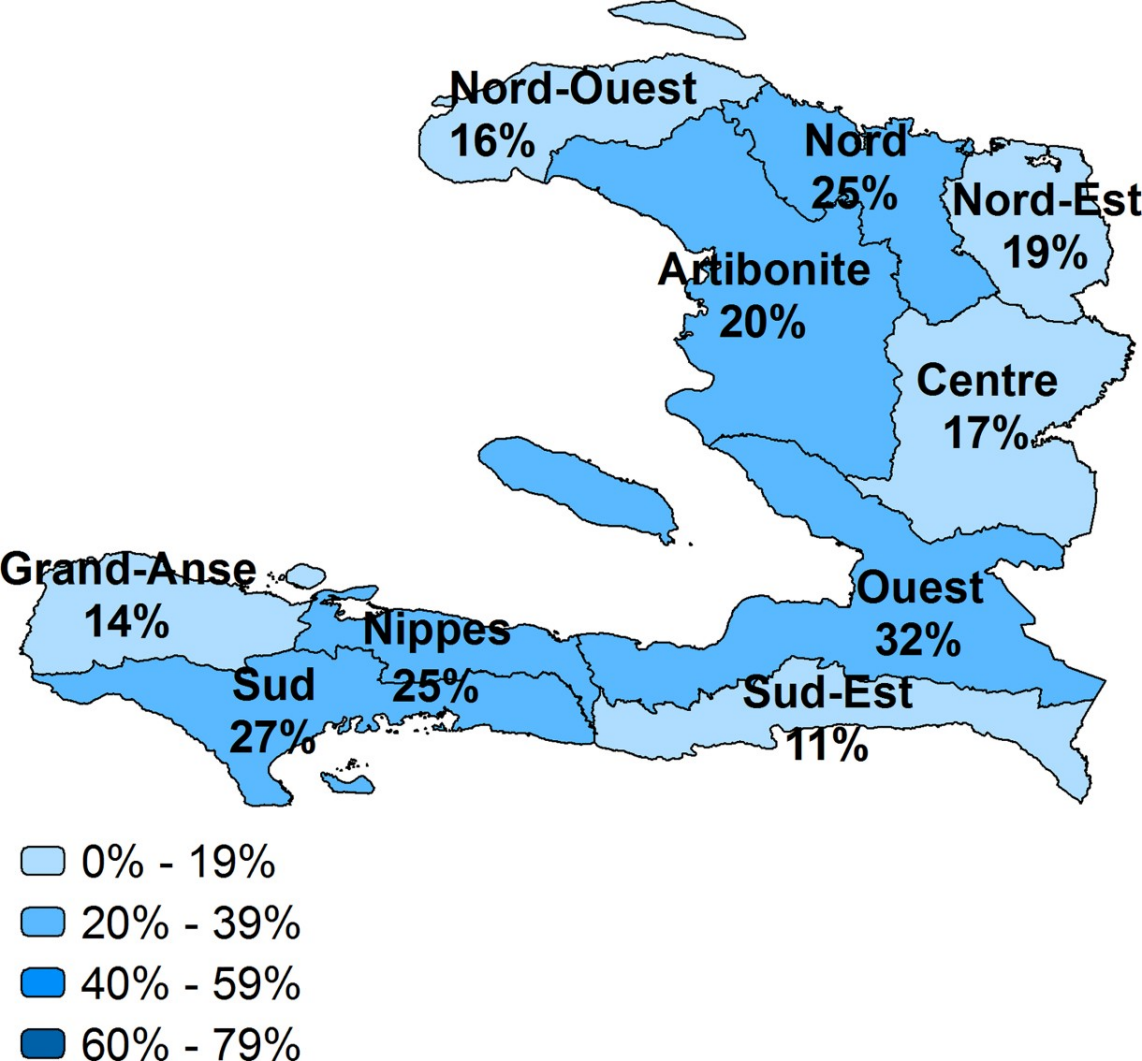
Effective coverage of facility delivery by region, Haiti.

**Fig 4:**
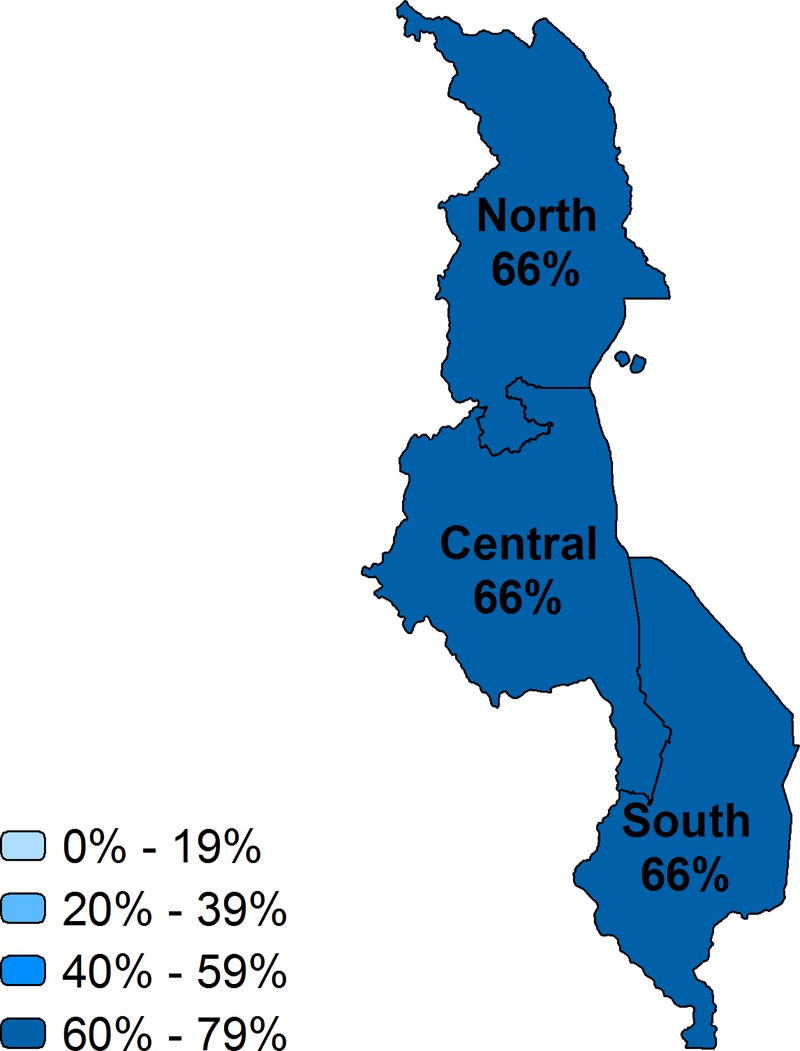
Effective coverage of facility delivery by region, Malawi.

**Fig 5:**
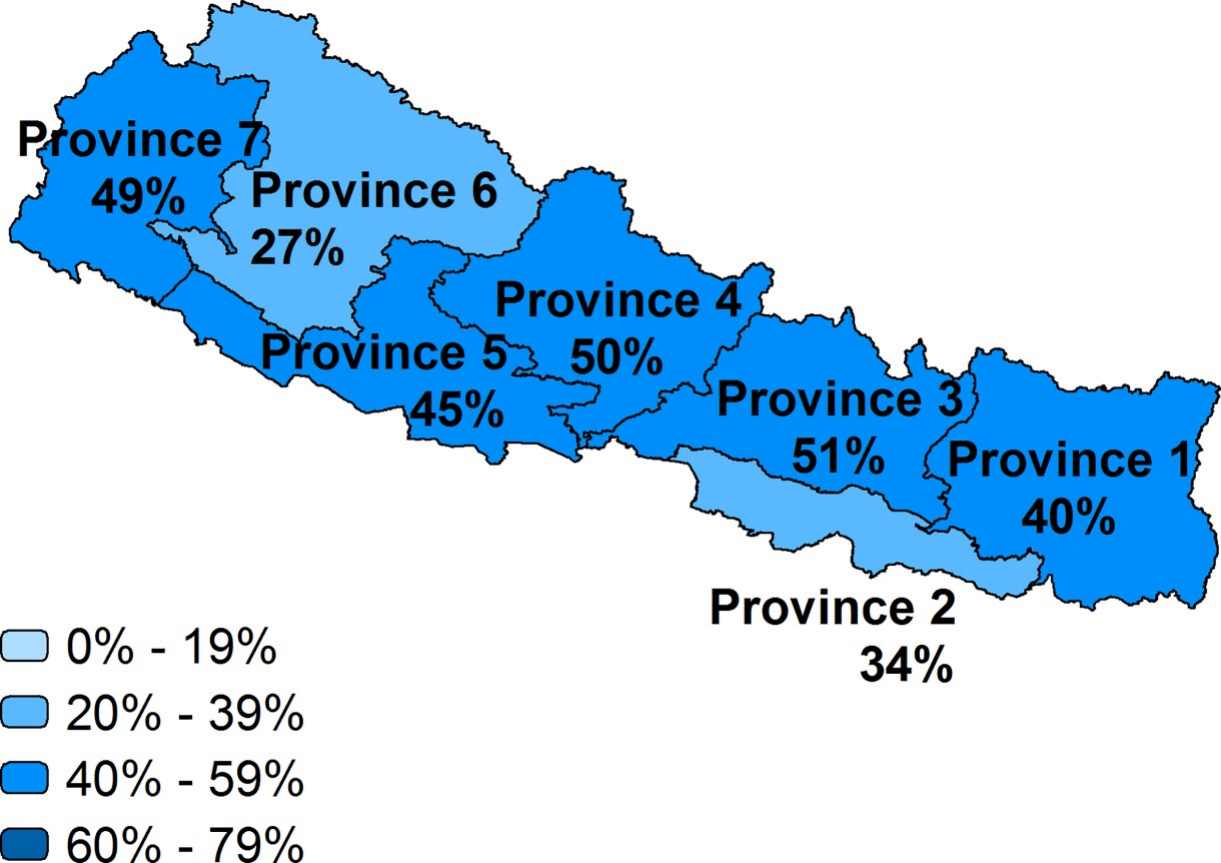
Effective coverage of facility delivery by region, Nepal.

**Fig 6:**
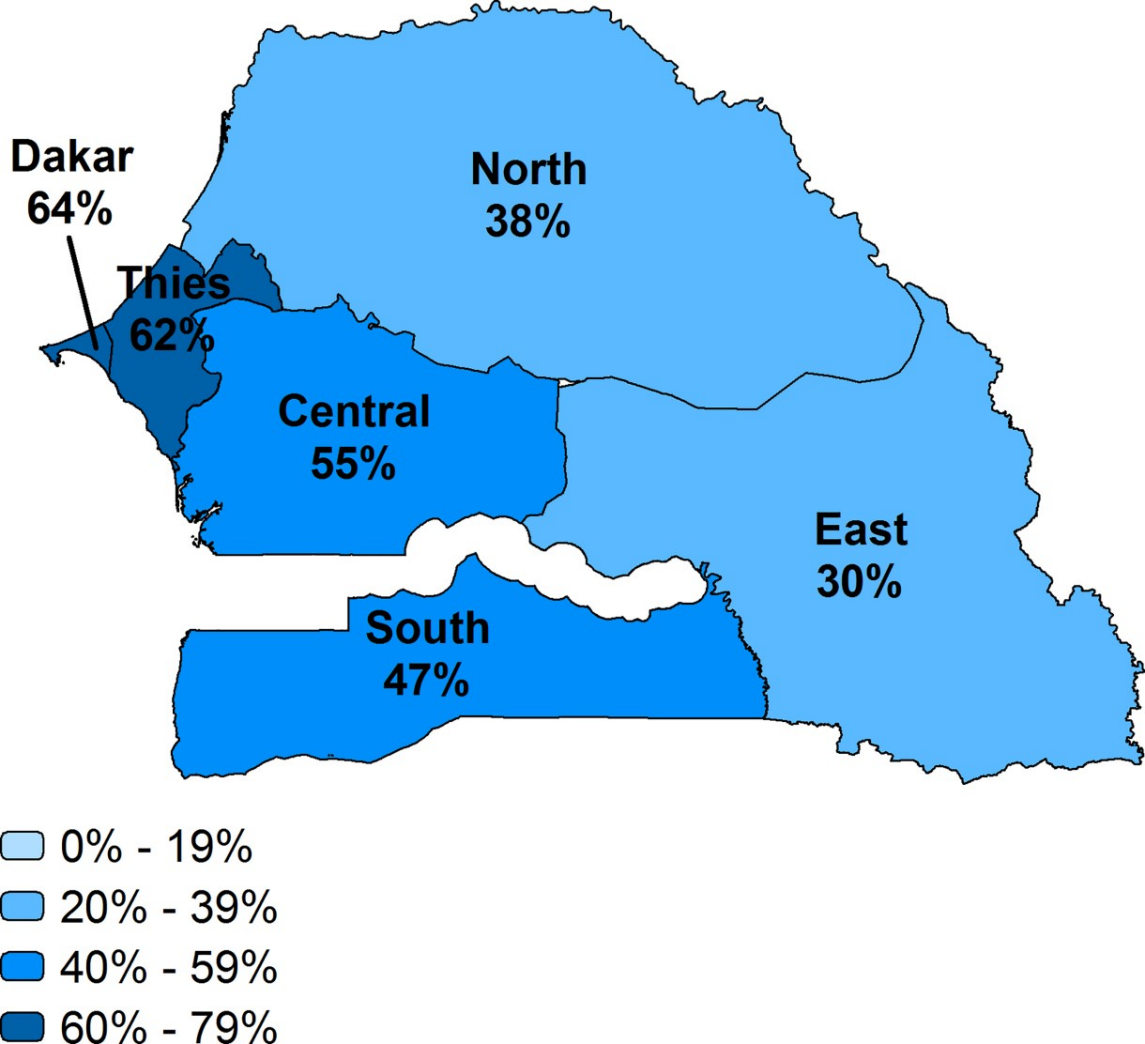
Effective coverage of facility delivery by region, Senegal.

**Fig 7:**
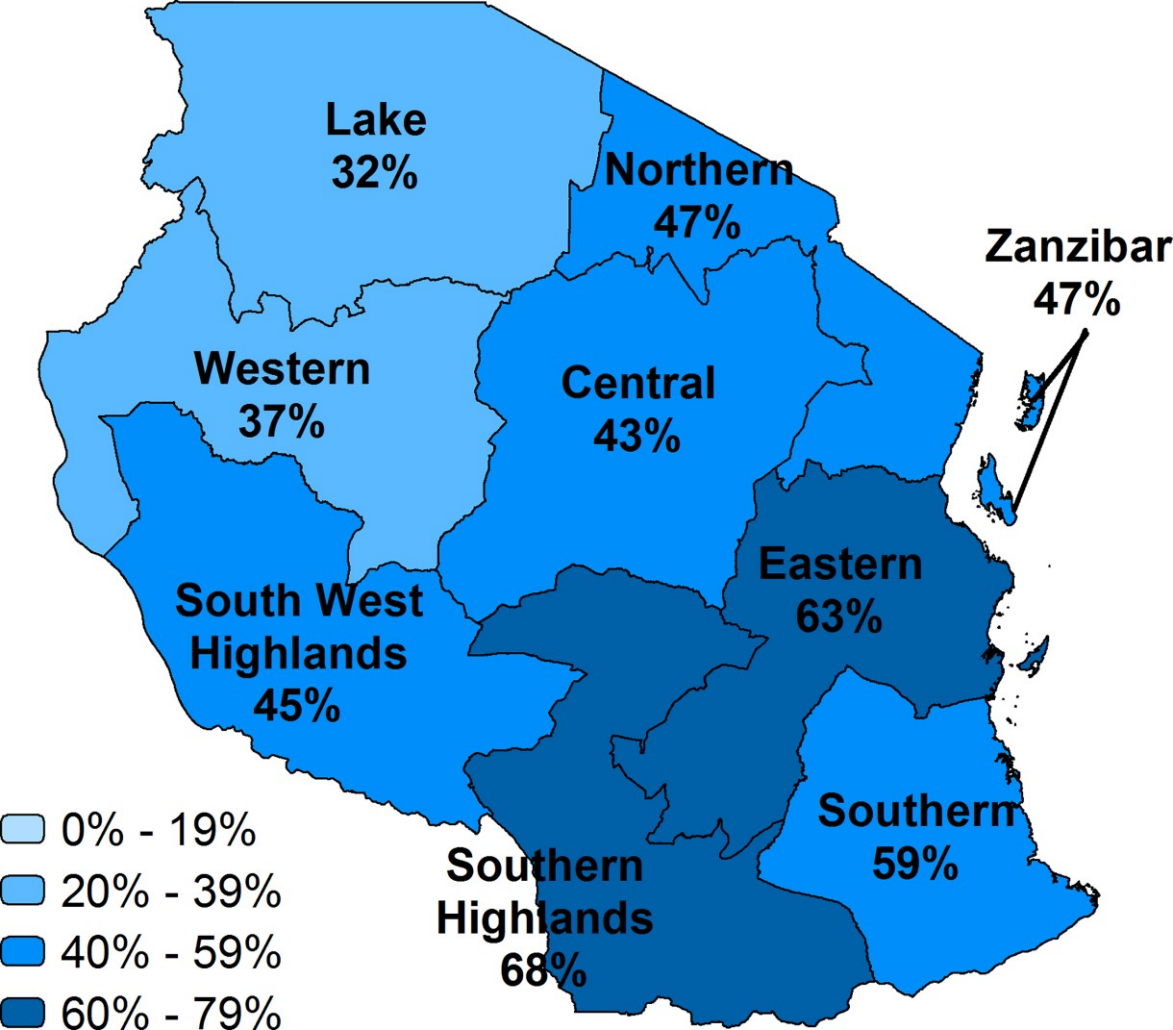
Effective coverage of facility delivery by region, Tanzania.

Taking into account the readiness of facilities to provide the service, we describe the effective coverage at the national and regional level in Figs 2–7 and Table 2. Malawi had the highest effective coverage, at 66%, 27 percentage points lower than its crude coverage. The effective coverage was 66% in each region. Senegal was the only other country with a national effective coverage above 50%, but with considerable variations among regions, from 30% in the East to 64% in Dakar. The level of effective coverage appeared lowest in the East (30%) and North (38%) compared with other regions. Across countries, the lowest effective coverage estimate was found in Haiti, at 24%, with effective coverage below 25% in most regions. Effective coverage was also low in Bangladesh, at 27%. Khulna had much higher effective coverage (41%) compared with all other divisions, while Sylhet had the lowest (16%); this score was significantly lower than in all other divisions except Barisal.

**Table 2:** Estimated regional and national effective coverage of facility delivery in all six countries.

	Coverage	Readiness score	Effective coverage		
			Estimate	LB	UB
**Bangladesh**					
Barisal	30.5	42.5	20.5	15.9	25.8
Chittagong	36.8	46.3	25.8	21.0	31.2
Dhaka	43.7	46.8	27.6	23.1	32.7
Khulna	58.8	49.3	40.9	35.0	47.0
Rajshahi	40.9	39.0	27.6	23.1	32.7
Rangpur	35.4	53.8	27.6	22.6	33.2
Sylhet	23.2	46.9	15.6	11.6	20.8
Total	39.7	46.5	26.8	24.5	29.1
**Haiti**					
Ouest	50.0	57.4	31.5	26.7	36.7
Sud-Est	23.0	47.0	10.5	6.3	17.0
Nord	39.9	54.0	25.3	18.6	33.3
Nord-Est	38.0	48.0	19.2	12.6	28.1
Artibonite	30.2	49.3	20.0	14.2	27.4
Centre	31.2	59.8	17.5	11.3	26.1
Sud	40.7	60.9	27.4	19.2	37.4
Grand-Anse	21.4	55.0	14.4	9.1	22.1
Nord-Ouest	31.5	43.2	16.1	11.2	22.5
Nippes	38.7	57.0	24.8	16.3	35.8
Total	40.0	52.7	24.4	22.0	27.0
**Malawi**					
North	94.7	67.5	66.2	58.2	73.4
Central	92.8	67.6	66.3	61.6	70.7
South	92.7	67.2	66.4	62.1	70.5
Total	92.9	67.4	66.4	63.4	69.2
**Nepal**					
Province 1	55.9	57.9	40.1	33.4	47.3
Province 2	37.4	63.0	33.7	28.4	39.5
Province 3	69.8	57.4	50.7	41.7	59.7
Province 4	67.1	54.6	50.3	41.7	58.8
Province 5	54.1	62.5	45.4	36.9	54.3
Province 6	34.1	52.4	27.1	21.4	33.6
Province 7	61.9	58.4	49.5	39.9	59.1
Total	52.7	57.7	41.9	38.9	45.1
**Senegal**					
North	62.9	54.3	37.8	31.7	44.4
Dakar	93.9	72.6	63.9	46.9	78.0
Thiès	91.9	56.9	61.6	48.8	73.0
Central	80.1	59.8	54.6	48.4	60.6
East	48.9	63.8	29.7	23.3	36.9
South	67.0	61.7	46.7	38.5	55.1
Total	77.0	60.0	51.3	47.2	55.3
**Tanzania**					
Western	53.0	56.0	37.0	28.9	46.0
Northern	68.2	58.7	46.8	37.9	55.8
Central	61.7	52.8	42.9	35.1	51.0
Southern Highlands	89.5	53.4	67.7	55.7	77.8
Southern	85.8	51.2	58.5	48.0	68.3
South West Highlands	69.3	52.7	45.2	36.2	54.5
Lake	50.6	48.0	32.4	28.5	36.6
Eastern	89.0	52.3	63.1	55.6	70.0
Zanzibar	70.2	55.3	47.1	42.5	51.8
Total	65.0	52.7	44.2	41.6	46.8

**Note:** LB and UB represent the lower and upper bounds of the 95% confidence interval of the effective coverage

While over half of births in Nepal were delivered in a health facility, the country’s effective coverage was 42%. Provinces 3, 4, and 7 had higher effective coverage than other provinces, about 50%; Provinces 6 and 2 had the lowest effective coverage, at 27% and 34% respectively. Nationally, effective coverage in Tanzania was 44%, which was substantially lower than the crude facility delivery coverage of 65%. There was a large variation in effective coverage by zones. The effective coverage in Southern Highlands (68%) was twice the level of the effective coverage in the Lake zone (32%).

## Discussion and conclusions

After taking into account facilities’ preparedness to provide delivery care services, the effective coverage in all countries studied is much lower than the crude coverage. The reduction ranges from 20% in Nepal to 39% in Haiti. Even though Malawi has almost universal facility delivery, the effective coverage is only 66%. Our results indicate that women who delivered in a health facility did not necessarily receive the quality of care needed to avoid preventable maternal and newborn mortality.[27] Taken along with findings from studies in other health areas and settings, [8, 28, 29] these highlight the need for improving quality of care to achieve the health-related Sustainable Development Goals.

A variety of facility types reported providing delivery care services, from lower-level, peripheral facilities to high-level facilities such as hospitals. Despite the poor readiness of the lower-level facilities, women often reported these facilities as the major sources of delivery care, as evidenced in Tanzania and Senegal. In Senegal, the most commonly used type of facility, the government health post, had low service readiness scores compared with government hospitals and health centers. Similarly in Tanzania, delivery care was commonly sought in public dispensaries, the facility type with the lowest readiness score. This was also identified by a study in a rural region of Tanzania, which found that while over 80% of women delivered in a health facility, few delivered at a facility that offered high-quality routine or emergency obstetric care.^7^. While lower-level facilities in the six countries were not equipped with all of the tracer items examined, many lacked essential supplies or equipment. A majority of non-CEmOC facilities lacked an emergency transportation system, which is critical for these facilities to be able to transfer medical emergencies or complications that they are not able to treat themselves. Even among high-level facilities, facilities are often poorly equipped. Staff training is important to ensure that health providers are technically competent, but in-service trainings are universally inadequate, indicating a need for more investment in human resources.[30, 31]

Haiti and Bangladesh have the lowest effective coverage among the six countries, resulting from both limited use of health facilities for delivery and poor readiness among the facilities. The destruction of health facilities in the 2010 earthquake, the mountainous terrain, and lack of high quality facilities in rural areas may inhibit equitable access to quality delivery services in Haiti.[32–34] Physical proximity to a health facility as well as quality of care provided at health facilities both play important roles in the use of services in Haiti.[35, 36] Similarly, in Bangladesh, among the many factors that could hinder women from using a health facility for delivery, the poor quality of services undoubtedly contributes to the low rate of use.[37] The poor quality of care in health facilities is believed to contribute to the stall of maternal mortality decline, despite an increase in facility delivery coverage.[38] Although private facilities generally provide better quality of care, they are usually less financially and geographically accessible than public facilities.[39, 40]

Senegal, Nepal, and Tanzania present intermediate levels of effective coverage, but demonstrate large regional variations, particularly in the coverage of facility delivery. In Senegal, for example, Dakar and Thiès, the two regions with the highest effective coverage, more than 90% of births were delivered in a health facility, while the East region had less than 50% facility delivery and suffered from the lowest effective coverage. In Nepal, the province with the highest percentages of facility deliveries had almost double the level of effective coverage compared with the province with the lowest percentages of facility deliveries. The finding that many women still delivery at home highlights the importance of continued efforts to improve the coverage of facility delivery in countries or regions where home delivery is still common. Malawi possesses the highest national crude delivery coverage and effective coverage. The high facility delivery coverage is due in part to a ban on informal birth attendants enacted in 2007—a policy aimed at transitioning births to the formal sector.[41] Additionally, adoption of the Newborn Action Plan prioritized quality of care during labor and delivery.[42]

This study is subject to several limitations. First, the effective coverage estimate is the facility delivery coverage adjusted for structural inputs. We did not assess process attributes, or providers’ adherence to acceptable standards of care. Facility readiness may not be indicative of provider performance. In fact, the association between structure and process was found to be weak.[43] Our results might overestimate effective coverage in the absence of data on the process of service delivery.

A scoring approach in measuring quality of care as used in this study is necessary to provide a comprehensive picture of a facility’s preparedness to provide delivery services and provide effective coverage estimates at the population level. That is, effective coverage aims to capture the expected level of coverage of services provided in a service delivery environment with optimal readiness. The readiness score itself cannot identify specific deficits. Facilities with a similar score could possess quite different specific tracer items. Effective coverage must be interpreted with pragmatism, and the tracer items used to compute the measure should always be referenced.

This study focused on estimating the effective coverage of facility delivery. It is important to note that some women who did not deliver in health facilities might still receive an adequate basic care from other sources such as trained traditional health attendant or community health workers. In fact, a randomized controlled trial in Pakistan showed that trained traditional birth attendants contributed to 30% reduction in perinatal mortality.[44] These non-institutional resources of care should be included when assessing the effective coverage of overall delivery care.[6]

Taking into account both crude coverage and quality of care, effective coverage is a useful tool for monitoring a country’s progress toward achieving universal coverage of health care with sufficient quality. We found that adjusting for facility readiness substantially reduces crude coverage of facility delivery. Such consistent findings in in all six countries reinforce the importance of prioritizing quality of obstetric and newborn care to achieve further reduction of maternal and neonatal mortality. Health care can only achieve its full potential when it offers sufficient quality. Meanwhile, continued efforts are needed to increase the use of facility delivery services in areas where coverage remains low.

## Supporting information

S1 Figs (PDF)

S1 Tables (PDF)

S1 File Description of variance estimation of effective coverage. (PDF)
