# Supplementary material for: Effective coverage of facility delivery in Bangladesh, Haiti, Malawi, Nepal, Senegal, and Tanzania
Source: PLoS One. 2019 Jun 11;14(6):e0217853. doi: 10.1371/journal.pone.0217853 (PMC6559642; doi:10.1371/journal.pone.0217853)

**Fig A. Distribution of facilities with delivery care by facility type**

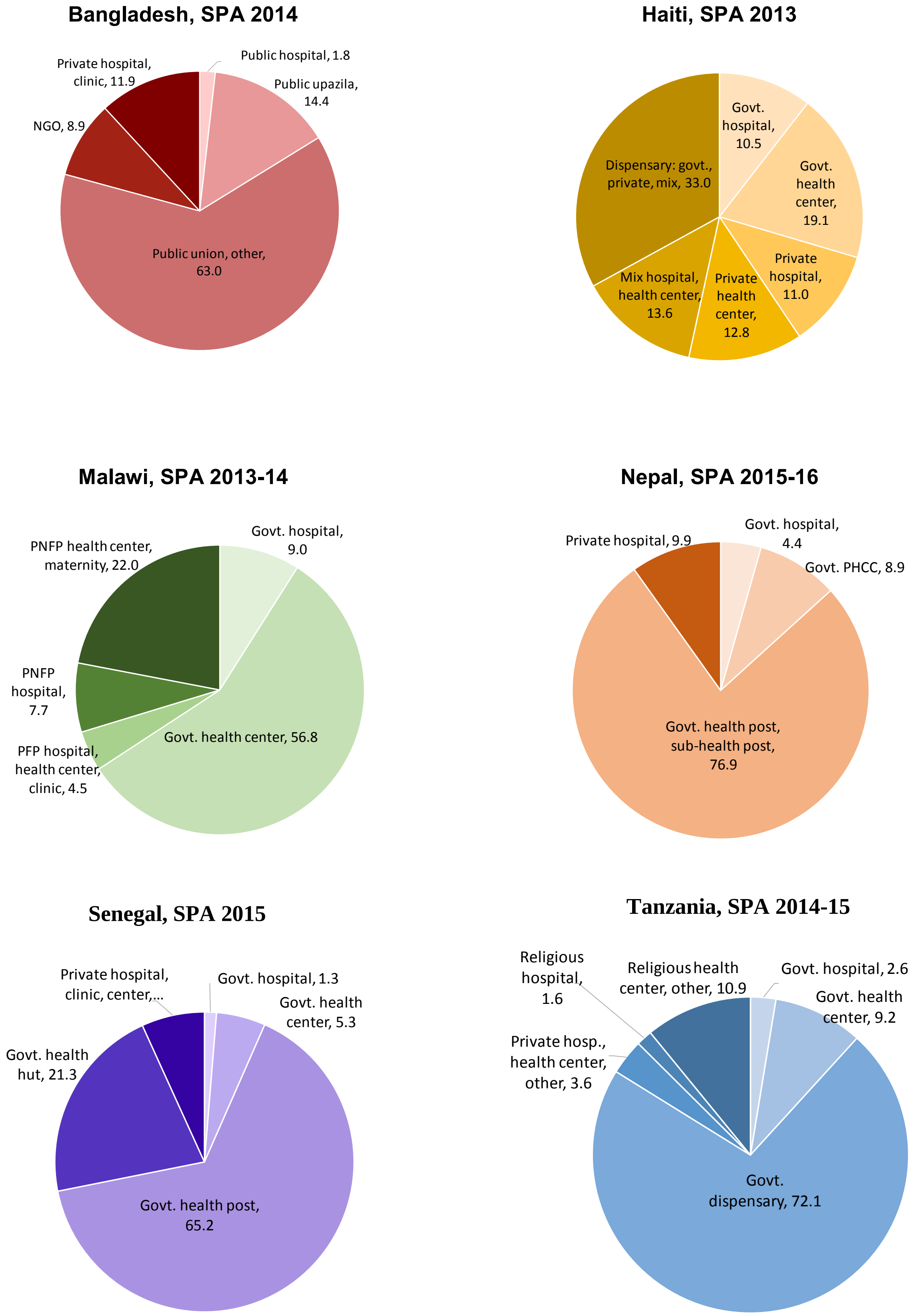

**Fig B. Readiness score of delivery services by facility type and division, Bangladesh SPA 2014**

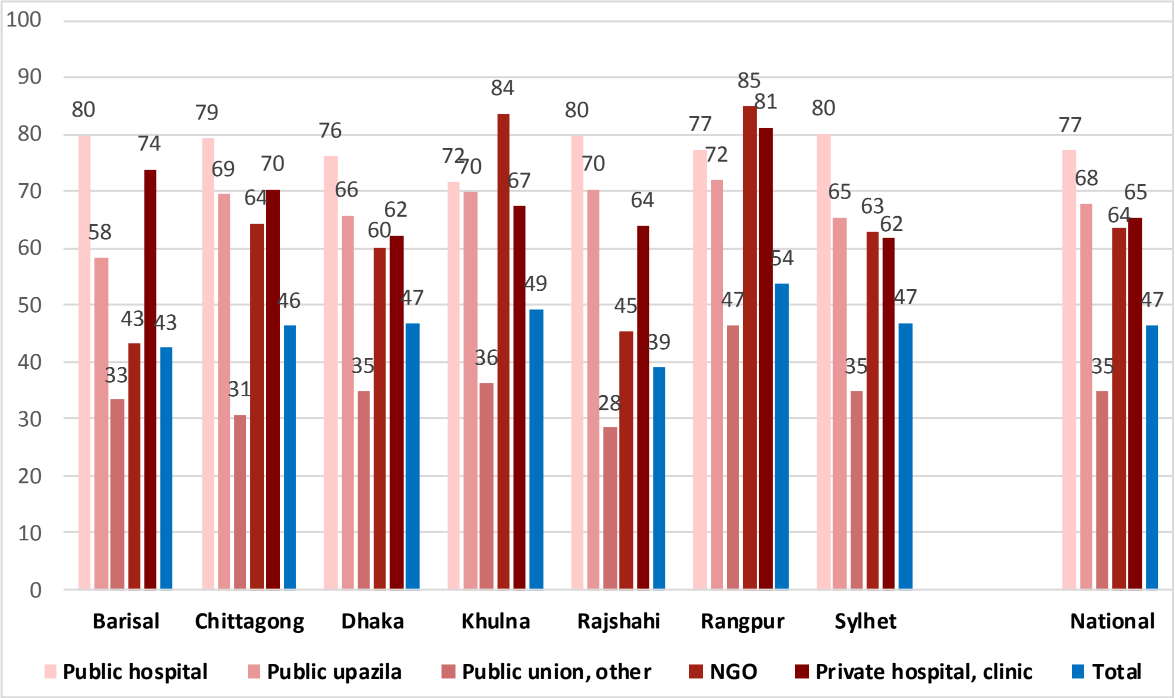


**Fig C. Percentage delivered in a health facility by facility type and division, among births in the two years preceding the survey, Bangladesh DHS 2014**

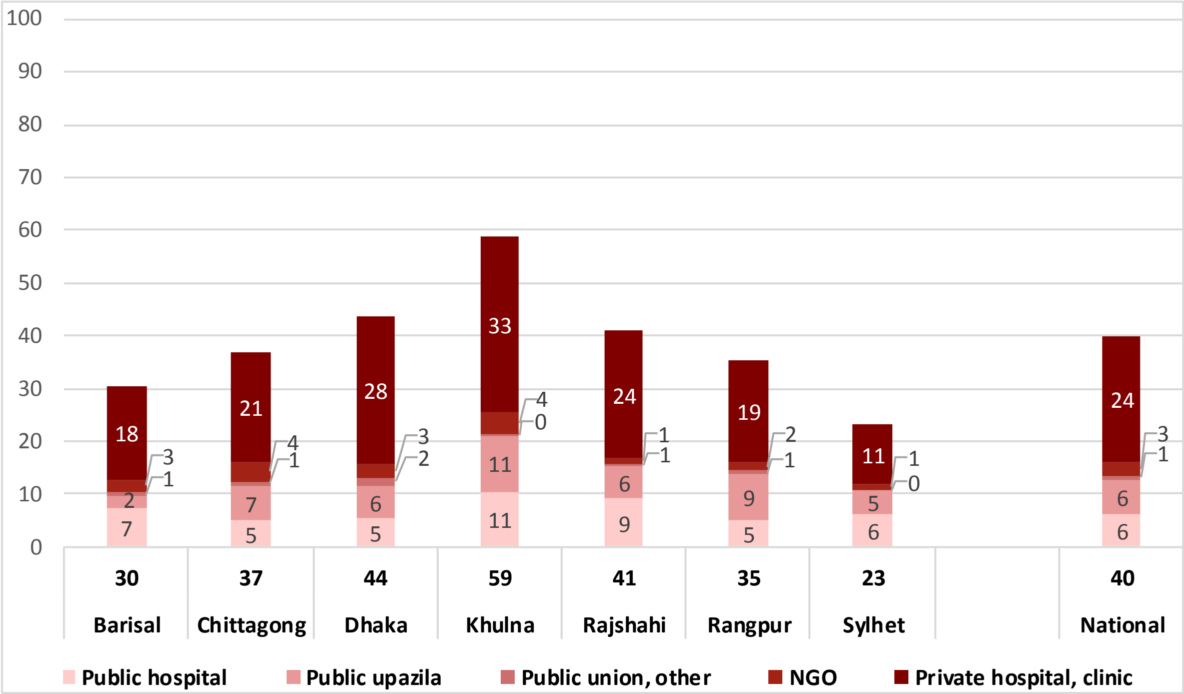

**Fig D. Readiness score of delivery services by facility type and region, Haiti SPA 2013**

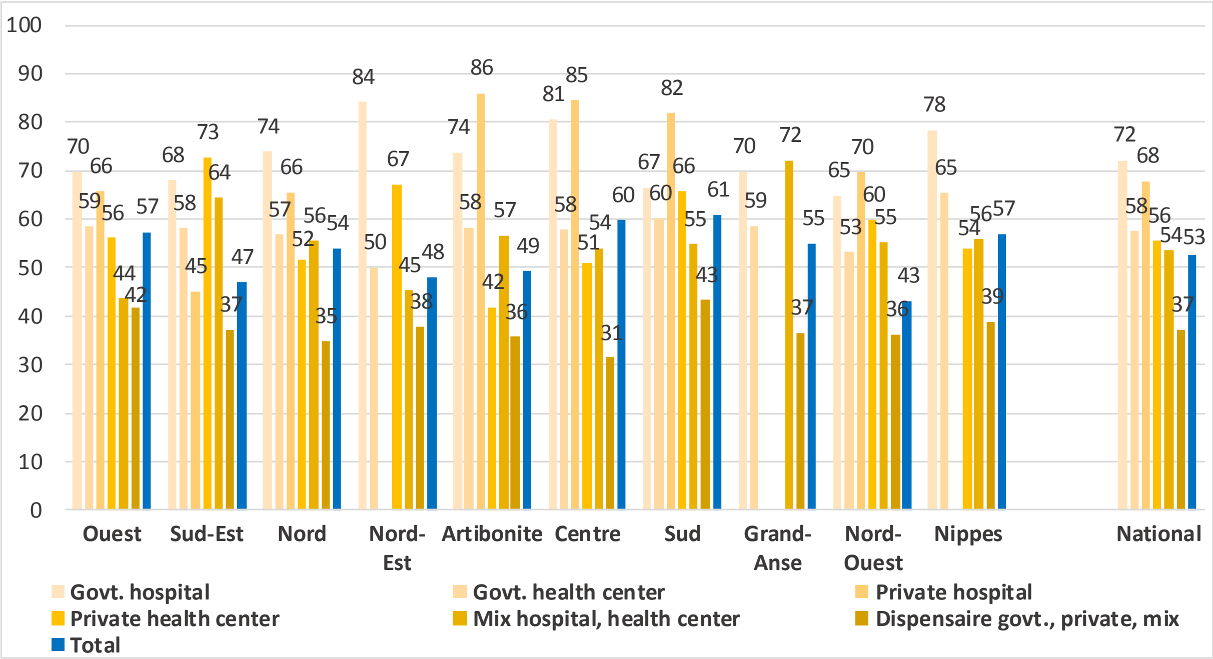


**Fig E. Percentage delivered in a health facility by facility type and region, among births in the two years preceding the survey, Haiti DHS 2012**

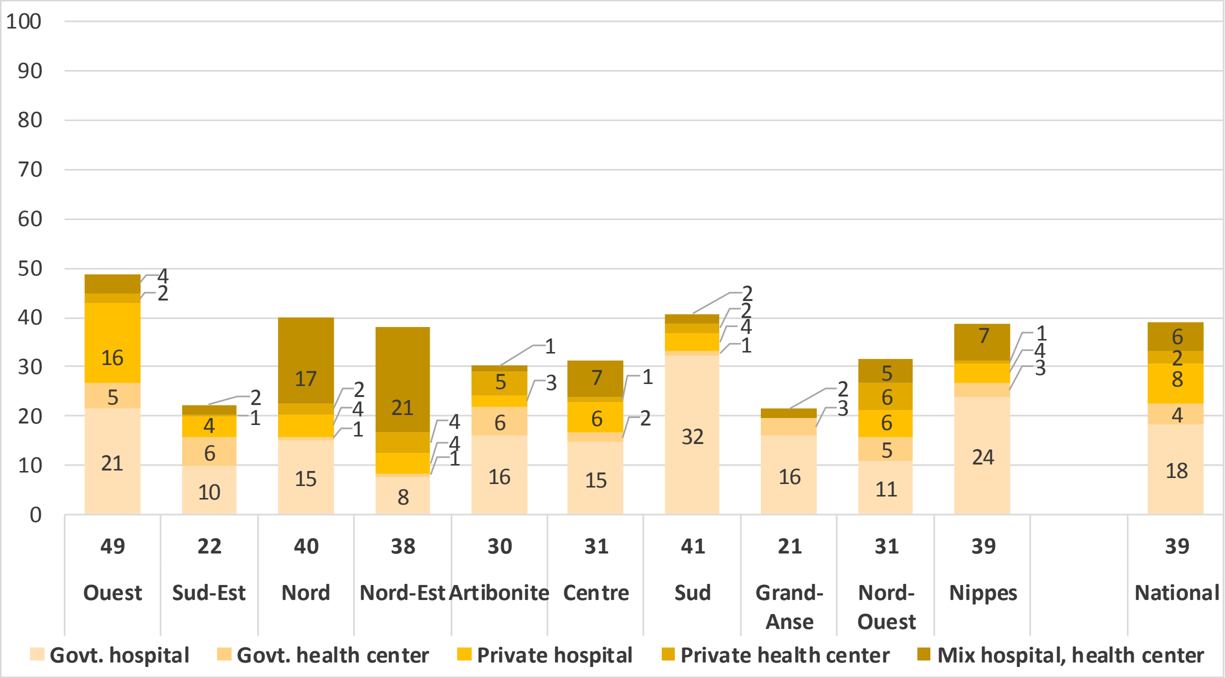

**Fig F. Readiness score of delivery services by facility type and region, Malawi SPA 2013-14**

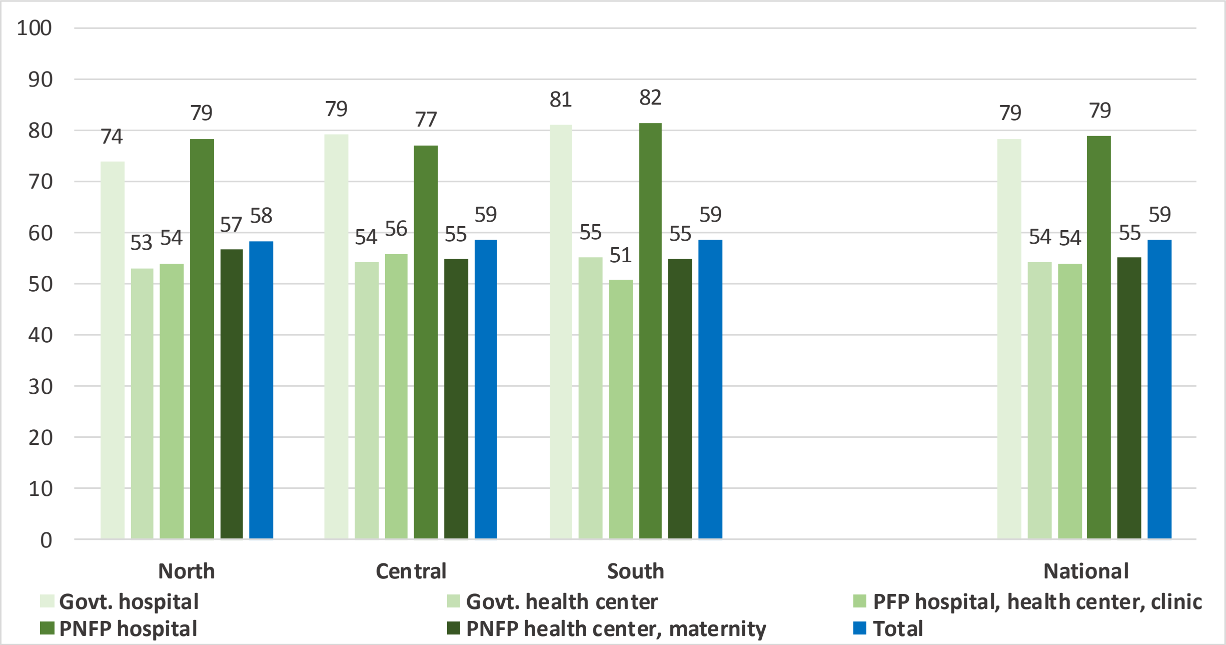


**Fig G. Percentage delivered in a health facility by facility type and region, among births in the two years preceding the survey, Malawi DHS 2012**

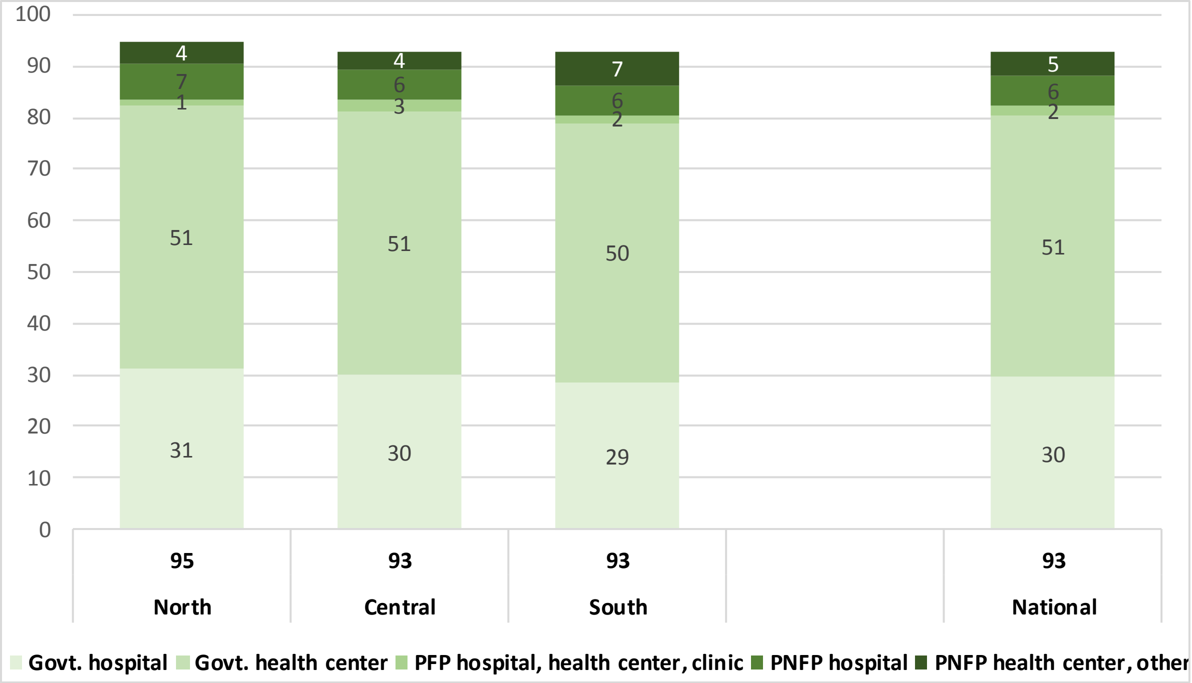

**Fig H. Readiness score of delivery services by facility type and province, Nepal SPA 2015-16**

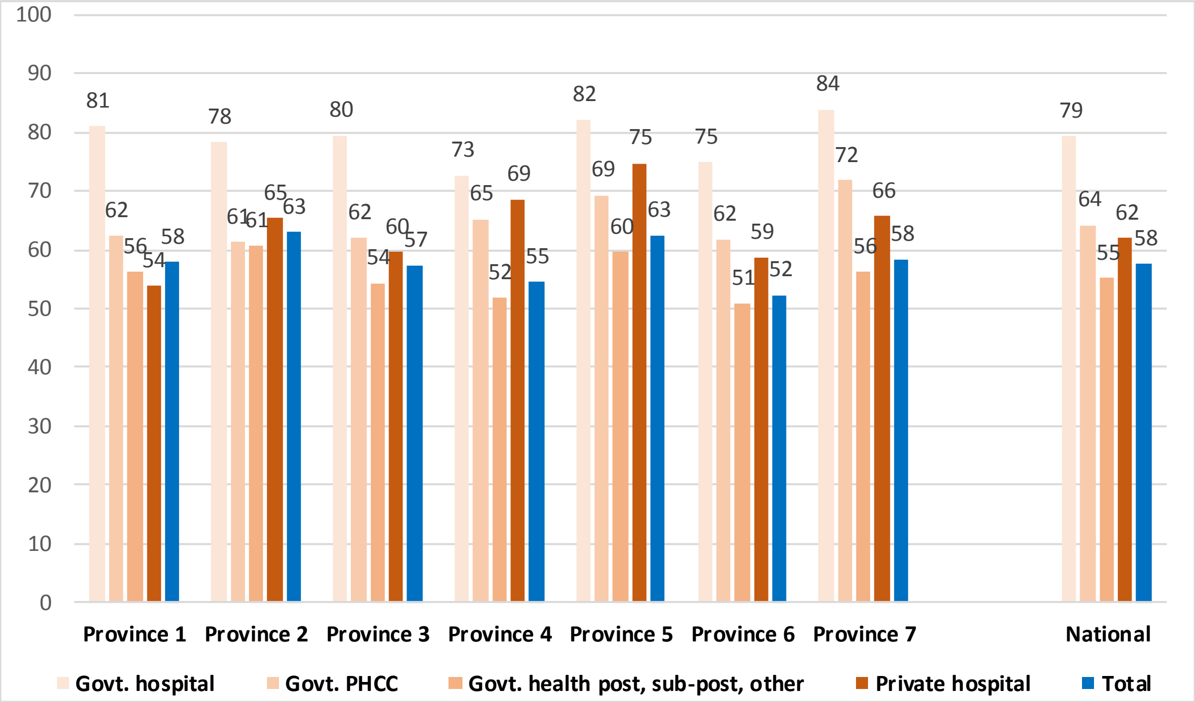


**Fig I. Percentage delivered in a health facility by facility type and province, among births in the two years preceding the survey, Nepal DHS 2015**

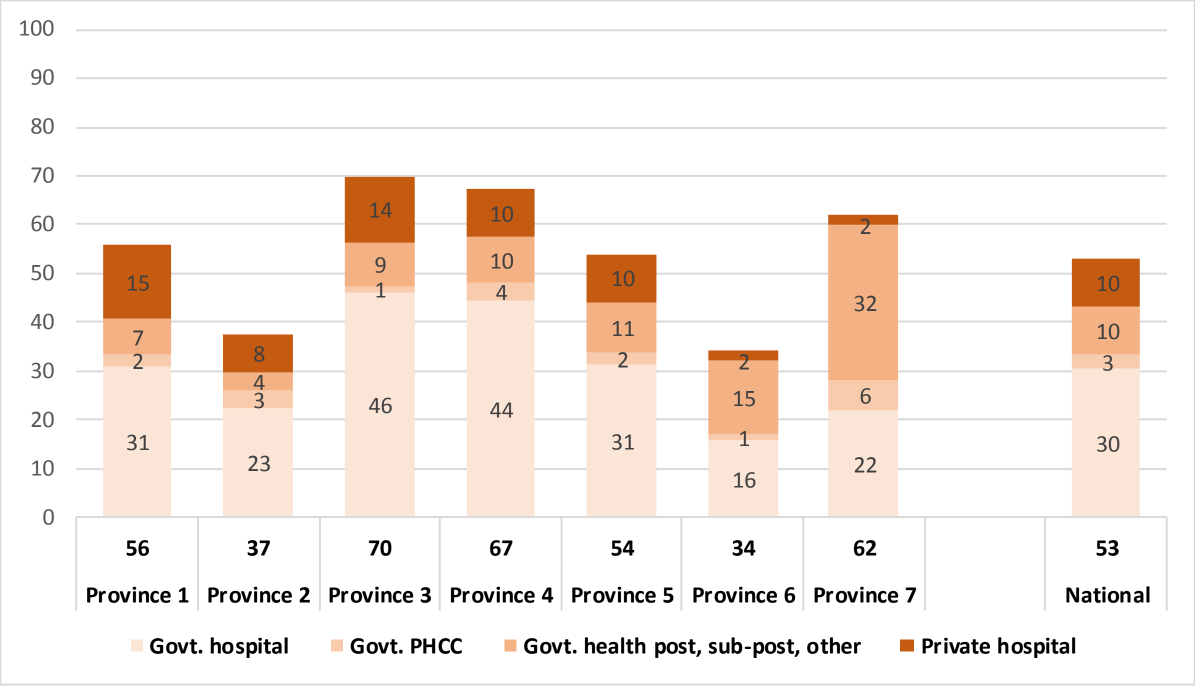

**Fig J. Readiness score of delivery services by facility type and region, Senegal SPA 2015**

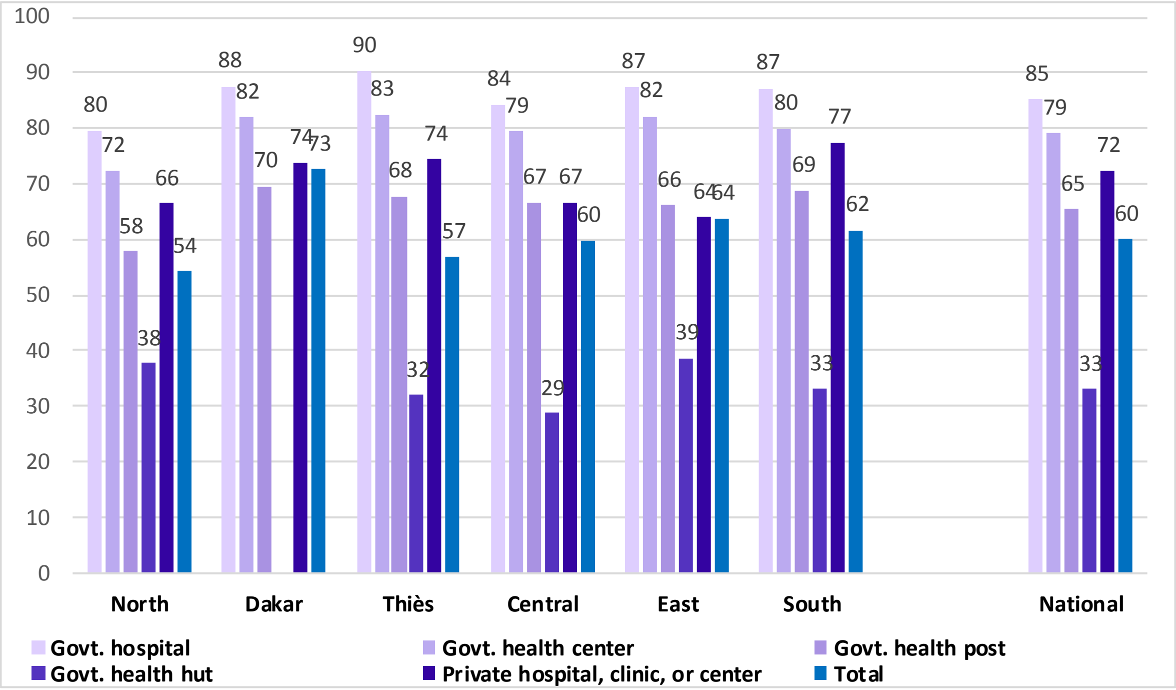


**Fig K. Percentage delivered in a health facility by facility type and region, among births in the two years preceding the survey, Senegal DHS 2016**

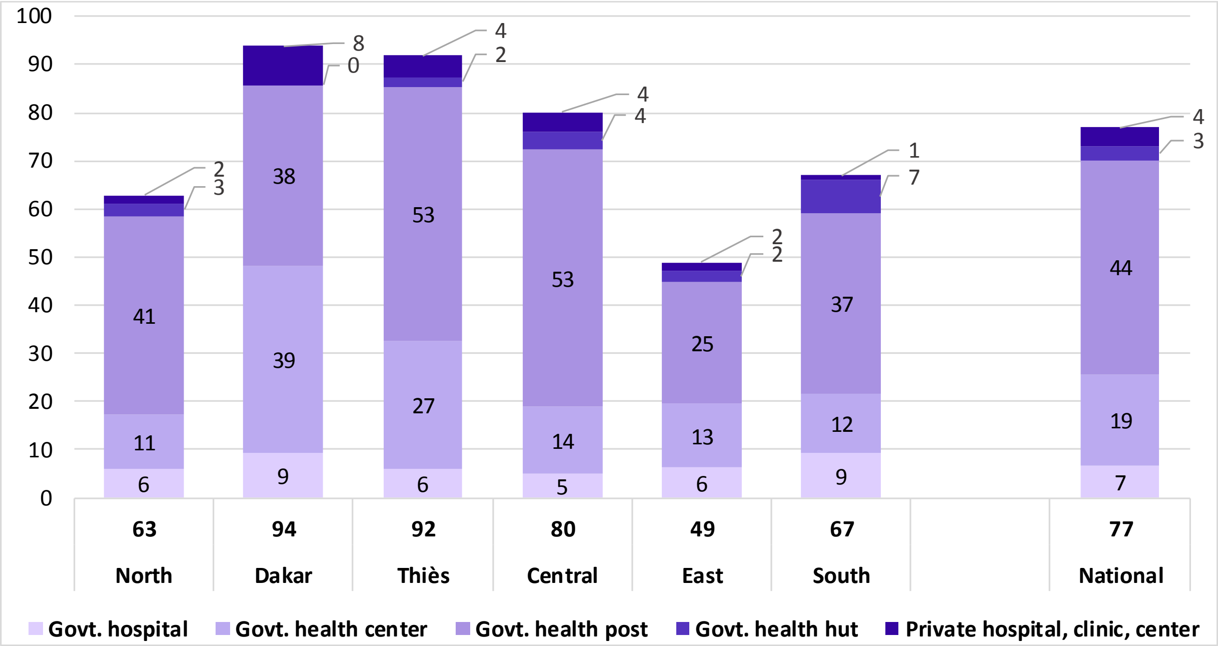

**Fig L. Readiness score of delivery services by facility type and zone, Tanzania SPA 2014-15**

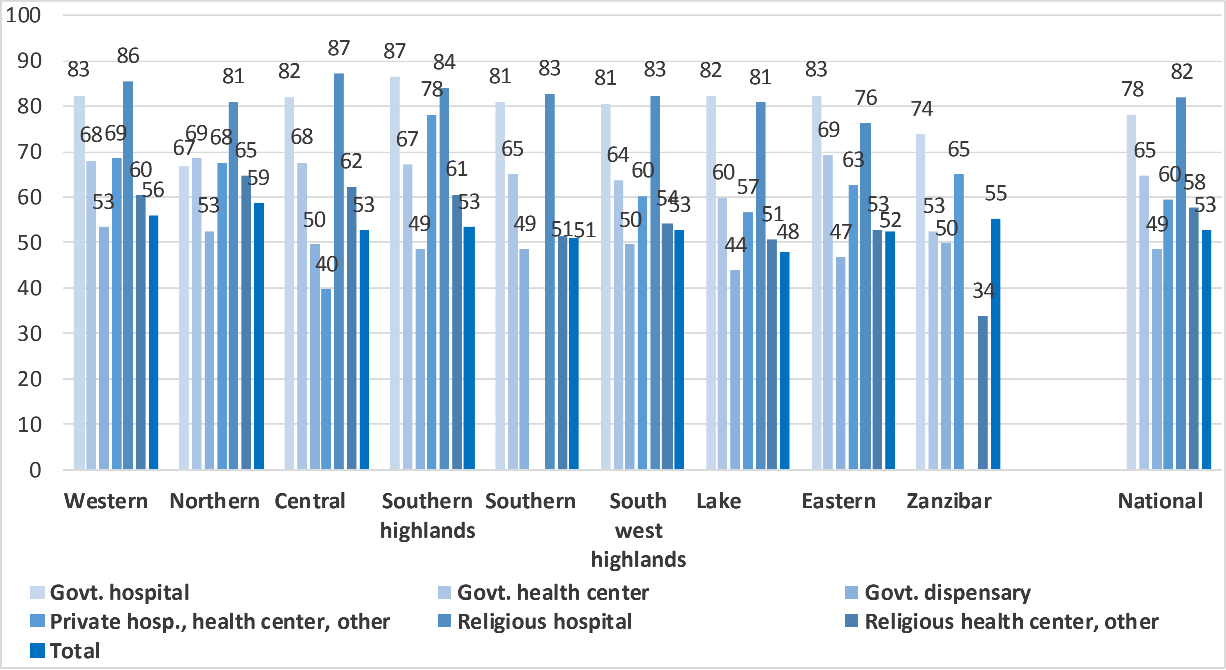


**Fig M. Percentage delivered in a health facility by facility type and zone, among births in the two years preceding the survey, Tanzania DHS 2015-16**

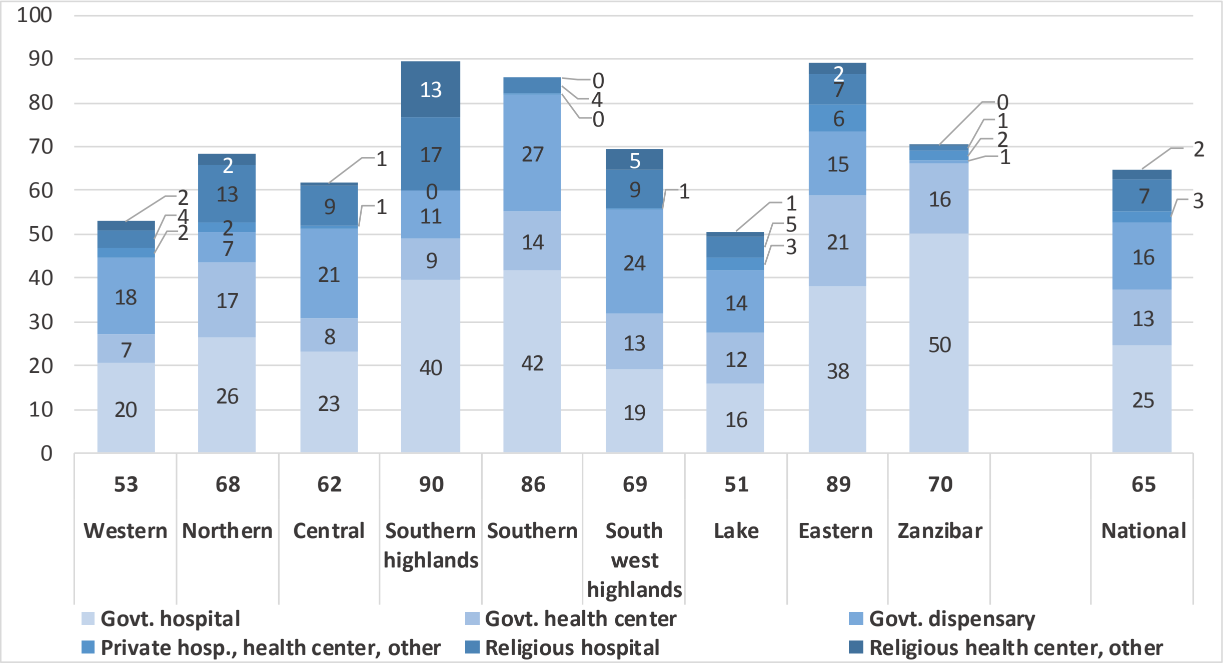

**Fig N. Readiness versus coverage by region**

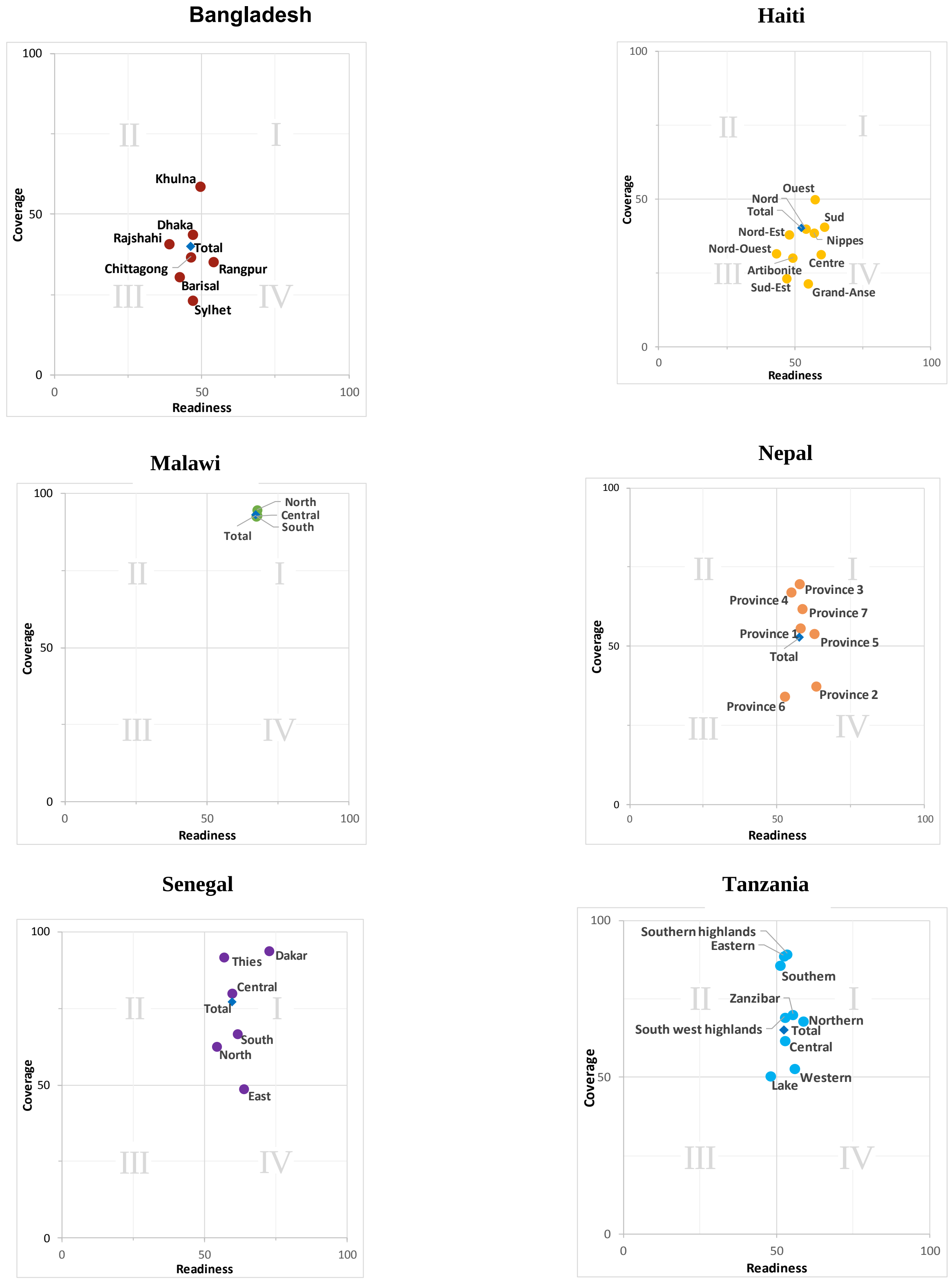

Supplement: S1 Figs — (PDF) [file pone.0217853.s001.pdf]
