# Supplementary material for: Effective coverage of facility delivery in Bangladesh, Haiti, Malawi, Nepal, Senegal, and Tanzania
Source: PLoS One. 2019 Jun 11;14(6):e0217853. doi: 10.1371/journal.pone.0217853 (PMC6559642; doi:10.1371/journal.pone.0217853)
Supplement: S1 Tables — (PDF) [file pone.0217853.s002.pdf]

Table A. Harmonized facility categories and reported categories in SPA and DHS

Harmonized facility category	SPA facility category	DHS facility category
**Bangladesh**		
Government hospital	Government district hospital	Government hospital<br>Government district hospital
Government upazila facilities[1]	Upazila health complex<br>Maternal and child welfare center	Upazila health complex<br>Upazila health & family welfare center<br>Maternal and child welfare center
Government union[2], other Government	Union health and family welfare center<br>Union health and family welfare center<br>Union subcenter (UNSC) / rural dispensary<br>Community clinic	Other Government sector<br>Community clinic
NGO	NGO clinic<br>NGO hospital	NGO clinic<br>Other NGO sector
Private hospital, clinic	Private hospital	Private hospital/clinic
**Haiti**		
Government hospital	Government university hospital<br>Government departmental hospital<br>Government community hospital<br>Other government hospital	Government hospital<br>Government maternity
Government health center	Government health center with bed<br>Government health center without bed	Government health center
Private hospital	Private university hospital<br>Private departmental hospital<br>private community hospital<br>Private hospital	Private hospital/clinic
Private health center	Private health center with bed<br>Private health center without bed	Private health center
Mix hospital, health center[3]	Mix hospital<br>Mix health center	Mix hospital<br>Mix health center<br>Mix maternity center
Dispensary	Dispensary	
**Malawi**		
Government hospital	Central hospital<br>District hospital<br>Rural/community hospital<br>Other hospital	Government hospital
Government health center	Government health center<br>Government maternity health	Government health center<br>Government health post<br>other Government sector
Private for-profit hospital, health center, clinic	Private hospital<br>Private health center<br>Private clinic<br>Private maternity center	Private for-profit hospital/clinic
Private not-for-profit hospital	private not-for-profit hospital	CHAM/mission hospital
Private not-for-profit health center, maternity	Private not-for-profit health center<br>Private not-for-profit maternity<br>Private not-for-profit clinic	CHAM/mission health center<br>BLM
**Nepal**		
Government Hospital	Central government hospital<br>Regional government hos<br>Sub-regional government hospital<br>Zonal government hospital<br>District government hospital	Government hospital
Government primary health care center (PHCC)	Government primary health care center (PHCC)	Government primary health care center (PHCC)
Government health post, sub-post, other	Government health post<br>Government sub-health post	Government health center<br>Other government sector
Private hospital	Private hospital	Private hospital
**Senegal**		
Government hospital	Government hospital	Government hospital
Government health center	Government health center	Government health center/maternity
Government health post	Government health center	Government health center

Government health hut[4]	Government health hut	Government health hut
Private hospital, clinic, or center	Private hospital<br>Private health center<br>Private health poster	Private hospital/clinic
**Tanzania**		
Government hospital	Government national referral hospital<br>Government regional hospital<br>Government district hospital<br>Government district-designated hospital<br>Other government hospital	Government national referral hospital<br>Government regional referral hospital<br>Government regional hospital<br>Government district hospital
Government health center	Government health center	Government health center
Government dispensary	Government dispensary	Government dispensary
Private hospital, health center, other	Private hospital<br>Private health center<br>private dispensary<br>private clinic	Private hospital<br>Private health center<br>private dispensary<br>private clinic
Religious hospital	Religious national referral hospital<br>Religious regional hospital<br>Religious district hospital<br>Religious district-designated hospital<br>Other religious hospital	Religious national referral hospital<br>Religious district hospital<br>Other religious hospital
Religious health center, other	Religious health center<br>Religious other	Religious health center<br>Religious other

[1]Pazila and union facilities represent facilities at the upazila and union administrative levels.

[2]Mix facilities in Haiti are non-profit facilities that also receive subsidies or salaried staff from the government.

Table B. Obstetric and newborn care readiness indicators and definitions

Domain/ Indicator Name	Definition
Domain A: Comprehensive emergency obstetric care	
Parenteral administration of antibiotics	Facility performed this signal function for emergency obstetric care at least once during the three months before the assessment
Parenteral administration of uterotonic drugs/oxytocin	See above
Parenteral administration of anticonvulsants for hypertensive disorders of pregnancy	See above
Manual removal of placenta	See above
Assisted vaginal delivery	See above
Removal of retained products	See above
Caesarean section	See above (incorporate the availability of equipment and materials for performing the service)
Blood transfusion	See above (incorporate the availability of equipment and materials for performing the service)
Domain B: Newborn signal functions and immediate care	
Neonatal resuscitation	Facility performed neonatal resuscitation at least once during the three months before the assessment
Skin-to-skin	Facility reported this intervention is routinely practiced
Breast feeding in 1st hour	See above
Drying and wrapping newborns	See above
Domain C: General requirements	
Electricity	Facility is connected to a central power grid and there has not been an interruption in power supply lasting for more than two hours at a time during normal working hours in the seven days before the assessment, or the facility had a functioning generator with fuel available on the day of the assessment, or else facility has a backup solar power.
Improved water source	Facility has an improved water source available. For most countries, this means that water is piped into the facility or onto facility grounds, or else water comes from a public tap or standpipe, a tube well or borehole, a protected dug well, protected spring, rain water, or bottled water, and the outlet from this source is within 500 meters of the facility.
Improved sanitation	Facility has a functioning flush or pour-flush toilet, a ventilated improved pit latrine, or composting toilet.
24/7 Skilled birth attendance	Provider of delivery care available on-site or on-call 24 hours/day, with observed duty schedule.
Emergency transport	The facility had a functioning ambulance or other vehicle for emergency transport that was stationed at the facility and had fuel available on the day of the assessment, or the facility has access to an ambulance or other vehicle for emergency transport that is stationed at another facility or that operates from another facility.
Domain D: Equipment	
Sterilization equipment	Facility reports that some instruments are processed in the facility and the facility has a functioning electric dry heat sterilizer, a functioning electric autoclave, or a non-electric autoclave with a functioning heat source available somewhere in the facility.
Delivery bed	At least one delivery bed available and observed in delivery area.
Examination light	Examination light (flashlight okay) available, observed, and functioning in delivery area.
Delivery pack	Delivery pack OR cord clamp, episiotomy scissors, scissors/lade to cut cord, suture material with need, AND needle holder all available in delivery area.
Suction apparatus (mucus abstractor)	Suction apparatus (mucus abstractor) available, observed, and functioning in the delivery area.
Manual vacuum extractor	Manual vacuum extractor available, observed, and functioning in the delivery area.
Vacuum aspirator or D&C kit	Vacuum aspirator or D&C kit available, observed, and functioning, in the delivery area.
Partograph	Partograph available, observed, and functioning in delivery area.
Disposable latex gloves	Disposable latex gloves observed in delivery area.
Newborn bag and mask	Newborn bag and mask (AMBU bag and mask) available, observed, and functioning in the delivery area.
Infant scale	Infant scale observed and functioning in delivery area.
Blood pressure apparatus (digital or manual)	Manual or digital blood pressure apparatus observed and functioning in delivery area.
Hand-washing soap and running water or hand disinfectant	Hand-washing soap and running water or hand disinfectant available and observed in delivery area.
Domain E: Medicines and commodities	
Injectable antibiotic	Injectable antibiotics observed in delivery area (i.e., at “service site”) and at least one dose valid.
Hydrocortisone available at the facility	Hydrocortisone observed at the facility and at least one dose valid.
Injectable uterotonic	Oxytocin observed in delivery area with at least one dose valid.
Skin disinfectant	Skin disinfectant available for newborns in delivery area.
Magnesium sulfate	Magnesium sulphate available in delivery area with at least one dose valid.
IV solution with infusion set	IV solution with infusion set available in delivery area with at least one set valid.
Chlorhexidine for cord cleaning	Chlorhexidine solution (4%) for umbilical cord cleaning available in delivery area, with at least one dose valid.
Antibiotic eye ointment for newborn	Tetracycline eye ointment for newborn available in delivery area and at least one dose valid.

Domain F: Guidelines, staff training and supervision	
Guidelines: Integrated Management of Pregnancy and Childbirth (IMPAC) Guidelines	Guidelines available in delivery area
Guidelines: CEmOC Guidelines	Guidelines available in delivery area
Guidelines: Guidelines for management of pre-term labor	Guidelines available in delivery area
Guidelines for standard precautions	Guidelines available in delivery area
Training in neonatal resuscitation	At least one provider of delivery/newborn care in facility received training in neonatal resuscitation in the past 24 months
Training in early and exclusive breastfeeding	At least one provider of delivery/newborn care in facility received training in early and exclusive breastfeeding in the past 24 months
Training in newborn infection management (including injectable antibiotics)	At least one provider of delivery/newborn care in facility received training in newborn infection management (including injectable antibiotics) in the past 24 months
Training in thermal care	At least one provider of delivery/newborn care in facility received training in thermal care in the past 24 months
Training in cord care	At least one provider of delivery/newborn care in facility received training in cord care in the past 24 months
Training in IMPAC	At least one provider of delivery/newborn care in facility received training in IMPAC in the past 24 months
Training in routine care during labor and delivery	At least one provider of delivery/newborn care in facility received training in routine care during labor and normal vaginal delivery in the past 24 months
Training in CEmOC	At least one provider of delivery/newborn care in facility received training in IMPAC in the past 24 months
Training in Active Management of Third Stage of Labor (AMTSL)	At least one provider of delivery/newborn care in facility received training in AMTSL in the past 24 months
Training in Kangaroo Mother Care (KMC)	At least one provider of delivery/newborn care in facility received training in KMC in the past 24 months
Supervision	At least half of interviewed providers reported being personally supervised at least once during the 6 months preceding the survey

Table C. Percentage of health facilities with structural tracer items, Bangladesh SPA 2014

Domain/Indicator Name	Non-CEmOC facilities								CEmOC facilities
	Barisal	Chittagong	Dhaka	Khulna	Rajshahi	Rangpur	Sylhet	National Average	National average
**Domain A: Comprehensive emergency obstetric care**									
Parenteral administration of antibiotics	46.1	49.1	46.5	38.6	28.2	37.1	36.1	42.2	100.0
Parenteral administration of uterotonic drugs/oxytocin	44.9	48.0	48.3	55.5	27.5	68.7	41.3	48.1	100.0
Parenteral administration of anticonvulsants for hypertensive disorders of pregnancy	17.2	21.0	23.6	31.1	17.6	53.1	28.4	26.5	100.0
Manual removal of placenta	43.8	41.9	35.7	34.4	39.7	45.4	43.3	39.4	100.0
Assisted vaginal delivery	40.1	43.0	46.1	39.8	46.1	43.9	63.0	45.4	100.0
Removal of retained products	27.8	27.3	25.8	25.5	29.7	43.5	28.3	29.0	100.0
Caesarean section	na	na	na	na	na	na	na	na	100.0
Blood transfusion	na	na	na	na	na	na	na	na	100.0
**Domain B: Newborn signal functions and immediate care**									
Neonatal resuscitation	29.8	34.2	45.0	55.9	26.9	48.9	31.1	40.2	94.3
Skin-to-skin care	51.6	57.3	70.5	84.4	62.4	90.9	62.5	69.0	93.1
Wrap baby	90.1	90.8	98.7	98.4	96.4	87.8	89.5	94.4	99.4
Initiate breastfeeding within the first hour	91.8	97.5	100.0	98.8	100.0	91.2	87.9	97.1	99.4
**Domain C: General requirements**									
Electricity	39.0	48.0	42.6	62.1	24.4	59.3	49.7	45.4	94.5
Improved water source	88.5	95.6	90.0	97.5	88.6	96.9	93.2	92.6	98.7
Improved sanitation	77.6	85.9	67.1	53.1	77.3	76.8	86.0	73.9	93.1
24/7 skilled birth attendance	37.8	25.7	27.6	30.7	17.4	44.0	30.1	28.9	64.7
Emergency transport	31.0	27.5	34.7	43.9	14.4	20.9	40.0	29.9	88.5
**Domain D: Equipment**									
Sterilization equipment	61.4	58.5	65.5	57.3	30.5	94.7	59.6	61.9	90.6
Delivery bed	80.2	67.9	76.7	64.9	70.9	68.8	73.4	72.2	92.4
Examination light	42.1	51.4	66.2	72.7	44.7	93.3	72.4	63.5	98.7
Delivery pack	54.1	47.9	59.5	44.4	56.9	89.7	52.1	58.4	82.0
Suction apparatus (mucus abstractor)	40.5	45.1	53.3	54.0	25.6	49.0	31.8	45.6	93.7
Manual vacuum extractor	22.6	28.1	18.1	18.6	14.8	40.1	25.9	23.2	52.7
Vacuum aspirator or D&C kit	27.9	33.9	20.1	32.0	10.5	45.5	29.2	26.8	66.3
Partograph	25.7	16.2	28.5	27.3	12.7	35.2	21.9	24.2	45.9
Disposable latex gloves	82.7	63.7	57.7	81.2	62.8	98.2	96.1	70.4	91.0
Newborn bag and mask	48.6	39.8	45.6	48.9	26.9	62.3	43.7	44.4	84.3
Infant scale	50.1	53.7	48.7	57.2	76.5	89.5	51.1	59.2	64.6
Blood pressure apparatus (digital or manual)	87.0	82.3	88.9	82.0	99.8	100.0	92.8	89.8	98.7
Hand-washing soap and running water or hand disinfectant	69.6	69.0	75.3	73.4	68.9	94.7	83.1	75.6	95.0
**Domain E: Medicines and commodities**									
Injectable antibiotic	20.1	30.4	39.8	18.8	27.6	23.2	30.0	30.8	68.4
Hydrocortisone available at the facility	11.2	20.7	21.7	13.5	8.8	11.7	20.5	17.3	71.6
Injectable uterotonic	19.9	26.9	33.6	30.2	28.1	46.2	33.4	32.0	79.1
Skin disinfectant	18.9	27.8	23.0	36.6	14.9	27.5	27.3	24.8	66.5
Magnesium sulfate	3.5	15.2	24.8	14.9	24.2	20.4	25.7	20.3	56.6
IV solution with infusion set	22.5	28.7	37.6	30.2	31.5	36.0	36.3	33.3	83.3
Chlorhexidine for cord cleaning	14.5	33.7	31.0	21.3	15.4	50.5	26.8	29.9	71.3
Antibiotic eye ointment for newborn	10.1	20.2	26.5	7.7	22.6	33.4	29.3	23.2	37.9

Domain F: Guidelines, staff training and supervision									
Guidelines: Integrated Management of Pregnancy and Childbirth (IMPAC) Guidelines	30.8	17.3	23.8	18.8	4.1	56.4	20.2	23.7	25.4
Guidelines: CEmOC Guidelines	16.4	22.5	15.1	20.9	9.8	56.7	20.7	21.9	28.9
Guidelines: Guidelines for management of pre-term labor	19.1	15.8	18.8	12.6	11.4	41.4	34.1	20.5	36.9
Guidelines on standard precaution	29.6	19.1	31.7	19.9	9.9	59.1	19.8	27.8	32.7
Training in neonatal resuscitation	19.3	24.7	11.8	48.4	6.7	17.4	43.4	20.1	29.9
Training in early and exclusive breastfeeding	18.8	28.3	16.7	35.3	2.2	19.7	39.4	20.8	21.7
Training in newborn infection management (including injectable antibiotics)	11.4	13.0	6.9	18.2	1.2	9.5	17.0	9.6	25.3
Training in thermal care	21.8	15.7	7.9	33.7	1.2	7.4	33.0	13.2	21.8
Training in cord care	21.8	19.4	13.5	43.8	2.2	10.5	28.7	17.0	29.2
Training in IMPAC	11.4	12.5	6.2	11.0	1.9	7.4	10.6	8.1	30.1
Training in normal labor and delivery care	17.8	13.4	7.6	18.9	3.1	7.7	17.8	10.4	33.3
Training in CEmOC	12.2	6.9	5.4	14.7	1.0	4.3	3.9	6.1	28.9
Training in AMTSL	16.9	15.8	5.4	23.6	1.2	8.4	15.1	10.2	32.0
Training in KMC	11.8	17.2	14.3	33.7	2.2	9.6	19.1	14.7	30.2
Supervision	82.4	89.2	87.9	89.3	90.3	95.2	84.0	88.9	92.0
Number of facilities	15	53	92	24	34	32	17	267	13

Table D. Percentage of health facilities with structural tracer items, Haiti SPA 2013

Domain/Indicator Name	Non-CEmOC facilities: Ouest	Sud-Est	Nord	Nord-Est	Artibonite	Centre	Sud	Grand-Anse	Nord-Ouest	Nippes	National Average	CEmOC facilities: National average
**Domain A: Comprehensive emergency obstetric care**												
Parenteral administration of antibiotics	70.3	40.0	61.1	42.3	45.1	47.6	73.9	40.0	46.1	50.0	54.6	100.0
Parenteral administration of uterotonic drugs/oxytocin	72.3	45.7	66.7	65.4	60.8	76.2	100.0	75.0	58.1	75.0	67.5	100.0
Parenteral administration of anticonvulsants for hypertensive disorders of pregnancy	34.7	17.1	36.1	23.1	29.4	28.6	34.8	35.0	21.8	18.8	29.0	100.0
Manual removal of placenta	55.4	34.3	47.2	42.3	52.9	42.9	56.5	65.0	42.2	50.0	49.3	100.0
Assisted vaginal delivery	79.2	65.7	80.6	73.1	62.7	76.2	87.0	70.0	63.9	81.3	73.3	100.0
Removal of retained products	53.5	48.6	50.0	42.3	41.2	52.4	47.8	65.0	32.0	37.5	46.9	100.0
Caesarean section	na	na	na	na	na	na	na	na	na	na	na	100.0
Blood transfusion	na	na	na	na	na	na	na	na	na	na	na	100.0
**Domain B: Newborn signal functions and immediate newborn care**												
Neonatal resuscitation	47.5	25.7	38.9	23.1	37.3	42.9	47.8	40.0	39.7	56.3	40.3	100.0
Skin-to-skin care	78.2	97.1	86.1	69.2	76.5	85.7	91.3	95.0	84.0	93.8	83.4	80.0
Wrap baby	97.0	97.1	94.4	84.6	88.2	95.2	100.0	95.0	96.0	100.0	94.7	100.0
Initiate breastfeeding within the first hour	86.1	85.7	97.2	84.6	88.2	95.2	100.0	90.0	96.0	93.8	90.5	90.0
**Domain C: General requirements**												
Electricity	83.2	68.6	88.9	69.2	62.7	85.7	95.7	95.0	74.0	68.8	78.4	90.0
Improved water source	72.3	85.7	77.8	69.2	68.6	71.4	87.0	85.0	70.1	93.8	75.5	80.0
Improved sanitation	78.2	28.6	36.1	38.5	41.2	52.4	47.8	35.0	15.9	56.3	47.1	50.0
24/7 skilled birth attendance	38.6	22.9	41.7	30.8	33.3	42.9	30.4	50.0	15.7	25.0	32.9	90.0
Emergency transport	40.6	25.7	30.6	11.5	41.2	42.9	34.8	15.0	7.9	31.3	30.0	80.0
**Domain D: Equipment**												
Sterilization equipment	55.4	40.0	50.0	53.8	35.3	76.2	56.5	25.0	35.9	50.0	47.5	100.0
Delivery bed	93.1	88.6	91.7	92.3	92.2	100.0	100.0	90.0	94.0	100.0	93.4	100.0
Examination light	46.5	28.6	27.8	38.5	27.5	42.9	47.8	45.0	23.9	43.8	36.6	30.0
Delivery pack	80.2	82.9	86.1	76.9	62.7	95.2	91.3	85.0	70.1	81.3	78.9	100.0
Suction apparatus (mucus abstractor)	45.5	11.4	25.0	15.4	25.5	14.3	43.5	35.0	11.9	18.8	27.7	10.0
Manual vacuum extractor	16.8	2.9	13.9	7.7	3.9	9.5	4.3	10.0	5.9	0.0	9.2	40.0
Vacuum aspirator or D&C kit	28.7	20.0	25.0	15.4	11.8	4.8	26.1	15.0	9.9	18.8	19.2	30.0
Partograph	22.8	25.7	19.4	23.1	23.5	28.6	26.1	40.0	7.9	31.3	22.7	90.0
Disposable latex gloves	93.1	94.3	88.9	96.2	92.2	90.5	87.0	100.0	81.9	93.8	91.3	100.0
Newborn bag and mask	49.5	25.7	38.9	15.4	27.5	47.6	56.5	25.0	11.8	43.8	34.8	80.0
Infant scale	73.3	74.3	91.7	65.4	60.8	90.5	78.3	75.0	65.9	62.5	72.8	90.0
Blood pressure apparatus (digital or manual)	87.1	88.6	91.7	73.1	84.3	100.0	82.6	75.0	92.1	93.8	87.1	70.0
Hand-washing soap and running water or hand disinfectant	80.2	71.4	63.9	65.4	64.7	81.0	69.6	50.0	65.9	75.0	70.4	60.0
**Domain E: Medicines and commodities**												
Injectable antibiotic	40.6	20.0	33.3	30.8	19.6	47.6	34.8	25.0	17.8	37.5	30.6	50.0
Hydrocortisone available at the facility	33.7	20.0	36.1	23.1	25.5	52.4	52.2	20.0	9.9	31.3	29.0	70.0
Injectable uterotonic	52.5	51.4	55.6	50.0	41.2	52.4	60.9	60.0	45.8	50.0	50.9	90.0
Skin disinfectant	66.3	51.4	61.1	65.4	64.7	76.2	73.9	65.0	53.7	68.8	63.5	90.0
Magnesium sulfate	44.6	28.6	36.1	34.6	33.3	61.9	52.2	45.0	25.8	25.0	38.2	100.0
IV solution with infusion set	40.6	40.0	50.0	50.0	41.2	52.4	34.8	40.0	31.8	43.8	41.4	60.0
Chlorhexidine for cord cleaning	43.6	51.4	47.2	46.2	39.2	42.9	65.2	45.0	26.0	62.5	44.0	40.0
Antibiotic eye ointment for newborn	45.5	40.0	63.9	42.3	58.8	57.1	52.2	40.0	41.9	37.5	48.3	80.0

**Domain F: Guidelines, staff training and supervision**												
Guidelines: Integrated Management of Pregnancy and Childbirth (IMPAC) Guidelines	15.8	31.4	19.4	26.9	23.5	19.0	21.7	15.0	26.0	25.0	21.7	60.0
Guidelines: CEmOC Guidelines	15.8	14.3	19.4	15.4	15.7	19.0	21.7	30.0	14.0	18.8	17.1	50.0
Guidelines: Guidelines for management of pre-term labor	12.9	5.7	16.7	3.8	11.8	23.8	13.0	20.0	9.9	18.8	12.6	20.0
Guidelines on standard precaution	8.9	5.7	25.0	7.7	11.8	4.8	4.3	10.0	9.9	37.5	11.3	60.0
Training in neonatal resuscitation	44.6	20.0	33.3	23.1	27.5	42.9	43.5	35.0	22.0	50.0	34.0	60.0
Training in early and exclusive breastfeeding	51.5	28.6	30.6	34.6	31.4	42.9	34.8	30.0	19.8	50.0	36.6	60.0
Training in newborn infection management (including injectable antibiotics)	39.6	22.9	25.0	23.1	31.4	42.9	26.1	25.0	18.0	37.5	30.1	30.0
Training in thermal care	43.6	31.4	27.8	26.9	25.5	42.9	34.8	35.0	17.8	50.0	33.2	70.0
Training in cord care	46.5	31.4	27.8	30.8	29.4	42.9	34.8	45.0	13.8	50.0	34.8	70.0
Training in IMPAC	43.6	31.4	30.6	42.3	35.3	52.4	47.8	20.0	26.0	43.8	37.2	70.0
Training in normal labor and delivery care	41.6	34.3	30.6	46.2	33.3	47.6	47.8	30.0	24.0	50.0	37.2	60.0
Training in CEmOC	37.6	25.7	27.8	34.6	27.5	42.9	34.8	20.0	20.0	43.8	31.1	60.0
Training in AMTSL	38.6	31.4	33.3	46.2	35.3	47.6	43.5	25.0	24.0	43.8	35.9	70.0
Training in KMC	33.7	20.0	25.0	23.1	25.5	42.9	30.4	20.0	5.9	37.5	25.8	50.0
Supervision	73.3	88.6	77.8	88.5	80.4	95.2	87.0	70.0	76.0	93.8	80.2	80.0
Number of facilities	100	35	36	26	51	21	23	20	51	16	379	10

Table E. Percentage of health facilities with structural tracer items, Malawi SPA 2013-14

Domain/Indicator Name	Non-CEmOC facilities North	Central	South	National Average	CEmOC facilities National average
**Domain A: Comprehensive emergency obstetric care**					
Parenteral administration of antibiotics	75.6	84.9	80.7	81.3	100.0
Parenteral administration of uterotonic drugs/oxytocin	98.1	97.0	98.7	97.9	100.0
Parenteral administration of anticonvulsants for hypertensive disorders of pregnancy	46.4	49.2	48.6	48.4	100.0
Manual removal of placenta	35.8	44.7	42.4	42.0	100.0
Assisted vaginal delivery	57.1	52.7	45.2	50.3	100.0
Removal of retained products	42.4	38.1	35.0	37.6	100.0
Caesarean section	na	na	na	na	100.0
Blood transfusion	na	na	na	na	100.0
**Domain B: Newborn signal functions and immediate newborn care**					
Neonatal resuscitation	93.2	90.5	82.8	87.7	100.0
Skin-to-skin care	100.0	96.5	98.7	98.1	100.0
Wrap baby	100.0	99.5	100.0	99.8	100.0
Initiate breastfeeding within the first hour	100.0	98.5	98.7	98.9	100.0
**Domain C: General requirements**					
Electricity	69.9	75.8	58.8	67.4	91.0
Improved water source	88.3	95.5	97.4	94.9	100.0
Improved sanitation	24.1	28.1	21.2	24.3	54.6
24/7 skilled birth attendance	36.6	52.2	60.9	52.8	100.0
Emergency transport	89.3	88.4	90.0	89.3	100.0
**Domain D: Equipment**					
Sterilization equipment	20.1	32.6	31.6	29.7	81.7
Delivery bed	98.0	99.0	98.3	98.5	100.0
Examination light	42.5	24.6	30.3	30.6	82.0
Delivery pack	86.4	90.4	96.0	92.0	100.0
Suction apparatus (mucus abstractor)	64.9	64.8	59.2	62.4	91.0
Manual vacuum extractor	34.7	43.1	38.5	39.5	100.0
Vacuum aspirator or D&C kit	18.2	23.1	24.5	22.7	91.0
Partograph	86.3	86.9	89.4	87.9	100.0
Disposable latex gloves	100.0	96.5	96.9	97.4	100.0
Newborn bag and mask	93.2	91.4	85.4	89.2	100.0
Infant scale	95.1	94.5	95.6	95.1	100.0
Blood pressure apparatus (digital or manual)	76.6	72.8	76.4	75.1	100.0
Hand-washing soap and running water or hand disinfectant	76.6	75.9	73.7	75.1	82.0
**Domain E: Medicines and commodities**					
Injectable antibiotic	57.2	52.2	55.3	54.5	100.0
Hydrocortisone available at the facility	13.4	9.5	13.3	11.9	73.0
Injectable uterotonic	90.3	97.0	95.6	95.1	100.0
Skin disinfectant	63.0	46.7	57.4	54.5	91.0
Magnesium sulfate	83.5	82.9	85.7	84.2	100.0
IV solution with infusion set	69.0	65.4	67.6	67.1	64.0
Chlorhexidine for cord cleaning	34.8	30.7	39.4	35.2	64.0
Antibiotic eye ointment for newborn	98.1	90.5	93.9	93.4	91.0
**Domain F: Guidelines, staff training and supervision**					
Guidelines: Integrated Management of Pregnancy and Childbirth (IMPAC) Guidelines	48.6	41.2	45.6	44.5	54.4
Guidelines: CEmOC Guidelines	40.8	21.6	25.0	26.8	54.7
Guidelines: Guidelines for management of pre-term labor	40.8	41.2	40.7	40.9	82.0
Guidelines on standard precaution	47.4	40.7	40.8	42.1	73.0
Training in neonatal resuscitation	70.7	58.2	60.4	61.6	91.0
Training in early and exclusive breastfeeding	56.2	45.2	45.6	47.5	72.9
Training in newborn infection management (including injectable antibiotics)	46.5	41.2	35.9	39.9	54.6
Training in thermal care	54.2	53.2	53.4	53.5	81.9
Training in cord care	55.2	55.7	53.4	54.6	72.9
Training in IMPAC	21.2	31.6	17.9	23.7	35.9
Training in normal labor and delivery care	37.7	44.7	32.8	38.2	54.3
Training in CEmOC	21.2	29.1	18.8	23.1	35.9
Training in AMTSL	35.7	47.7	34.5	39.7	54.3
Training in KMC	42.6	43.2	35.9	40.0	54.3
Supervision	76.7	86.4	82.9	83.0	91.0
Number of facilities	102	194	221	517	11

Table F. Percentage of health facilities with structural tracer items, Nepal SPA 2015

	Non-CEmOC facilities								CEmOC facilities
**Domain/Indicator Name**	Province 1	Province 2	Province 3	Province 4	Province 5	Province 6	Province 7	National Average	National average
**Domain A: Comprehensive emergency obstetric care**									
Parenteral administration of antibiotics	35.6	64.6	40.2	35.5	57.8	28.6	33.5	39.5	100.0
Parenteral administration of uterotonic drugs/oxytocin	83.1	95.7	77.3	76.0	93.8	85.3	96.2	85.5	100.0
Parenteral administration of anticonvulsants for hypertensive disorders of pregnancy	7.9	27.0	9.9	4.1	15.8	5.5	6.8	8.1	100.0
Manual removal of placenta	51.3	54.3	31.6	32.4	57.1	34.4	44.7	41.6	100.0
Assisted vaginal delivery	11.4	35.0	12.9	8.7	22.4	16.5	15.3	14.3	100.0
Removal of retained products	40.5	44.3	25.6	15.5	46.0	23.9	39.8	31.6	100.0
Caesarean section	na	na	na	na	na	na	na	na	100.0
Blood transfusion	na	na	na	na	na	na	na	na	100.0
**Domain B: Newborn signal functions and immediate newborn care**									
Neonatal resuscitation	42.3	54.0	27.9	19.1	46.1	43.5	33.5	35.5	97.8
Skin-to-skin care	96.4	84.9	89.0	98.1	93.3	75.5	93.5	90.8	85.4
Wrap baby	97.2	100.0	100.0	97.9	100.0	86.1	100.0	97.3	100.0
Initiate breastfeeding within the first hour	98.5	100.0	99.7	99.7	100.0	94.1	100.0	98.8	100.0
**Domain C: General requirements**									
Electricity	80.5	69.0	72.4	54.0	77.1	94.2	76.3	74.5	100.0
Improved water source	91.8	100.0	91.9	87.6	85.2	61.9	77.7	84.7	95.6
Improved sanitation	97.1	90.6	95.3	92.0	89.8	79.4	83.3	89.9	97.8
24/7 skilled birth attendance	21.5	39.3	30.4	27.3	21.3	5.3	22.1	22.4	70.6
Emergency transport	55.1	81.7	81.1	67.4	64.1	37.7	51.7	61.5	97.8
**Domain D: Equipment**									
Sterilization equipment	95.0	91.9	96.6	91.4	88.6	81.7	95.3	91.8	97.6
Delivery bed	94.9	96.2	95.0	95.8	99.7	93.3	100.0	96.3	97.6
Examination light	60.8	65.3	68.2	73.7	63.5	38.1	54.2	59.9	97.6
Delivery pack	91.4	95.2	91.1	87.5	100.0	91.5	95.1	92.8	97.6
Suction apparatus (mucus abstractor)	63.0	87.8	76.9	77.0	52.0	31.5	50.0	61.2	97.6
Manual vacuum extractor	28.2	28.8	22.0	13.4	16.9	14.1	22.6	19.3	87.4
Vacuum aspirator or D&C kit	18.4	35.0	25.1	10.4	17.7	10.8	21.3	17.9	80.8
Partograph	68.3	72.4	80.2	93.8	91.1	82.7	71.6	79.9	85.1
Disposable latex gloves	96.0	96.5	91.1	95.7	95.4	85.9	88.3	92.6	91.8
Newborn bag and mask	80.9	93.6	89.6	72.7	88.2	71.5	85.7	82.5	97.6
Infant scale	80.5	93.8	87.3	87.0	99.7	88.2	96.7	89.7	95.4
Blood pressure apparatus (digital or manual)	78.4	76.9	81.5	87.4	90.7	80.3	84.2	82.6	97.6
Hand-washing soap and running water or hand disinfectant	69.8	78.2	84.2	84.3	81.1	61.0	61.3	74.0	87.4
**Domain E: Medicines and commodities**									
Injectable antibiotic	41.6	62.3	42.6	30.4	48.6	39.0	30.2	39.8	90.9
Hydrocortisone available at the facility	10.9	22.4	25.3	17.5	13.8	9.1	14.0	14.9	70.3
Injectable uterotonic	83.8	92.2	82.5	89.7	95.5	78.7	98.0	88.0	97.6
Skin disinfectant	87.5	96.2	94.7	91.4	93.9	83.7	93.4	91.2	97.6
Magnesium sulfate	62.9	59.8	63.1	84.0	81.9	65.3	86.7	71.6	97.6
IV solution with infusion set	88.0	89.8	86.8	93.6	92.9	83.2	98.6	90.4	87.4
Chlorhexidine for cord cleaning	61.9	52.6	60.4	41.0	75.7	37.6	72.3	58.3	41.8
Antibiotic eye ointment for newborn	25.1	24.5	40.0	53.4	48.2	45.2	37.0	40.0	13.3

Domain F: Guidelines, staff training and supervision									
Guidelines: Integrated Management of Pregnancy and Childbirth (IMPAC) Guidelines	na	na	na	na	na	na	na	na	na
Guidelines: CEmOC Guidelines	na	na	na	na	na	na	na	na	na
Guidelines: Guidelines for management of pre-term labor	na	na	na	na	na	na	na	na	na
Medical Standards Volume III or reproductive health Guidelines	19.5	20.4	9.5	25.9	41.6	20.4	19.2	22.0	13.1
Guidelines on standard precaution	1.6	4.3	3.9	14.8	11.8	5.2	8.7	6.9	14.6
Training in neonatal resuscitation	30.7	25.5	25.6	12.0	30.6	44.6	34.9	29.2	26.9
Training in early and exclusive breastfeeding	32.6	24.5	23.3	13.5	30.6	52.4	32.9	30.1	22.1
Training in newborn infection management (including injectable antibiotics)	23.5	8.5	11.6	6.5	23.3	38.9	19.0	19.2	11.0
Training in thermal care	30.7	21.4	21.4	11.4	26.2	42.4	27.7	26.2	15.3
Training in cord care	30.7	25.5	21.2	10.8	31.2	38.5	34.4	27.5	17.5
Training in IMPAC	2.0	3.8	0.0	0.3	0.0	0.7	0.6	0.9	2.2
Training in normal labor and delivery care	30.6	24.6	28.6	13.5	25.3	23.9	30.2	25.5	29.9
Training in CEmOC	13.1	8.5	7.0	6.5	13.3	15.2	20.4	12.1	11.0
Training in AMTSL	31.9	33.7	29.1	12.9	24.1	26.2	32.4	27.0	29.9
Training in KMC	30.9	19.2	21.7	13.8	32.5	40.8	31.7	27.6	19.7
Supervision	76.0	81.6	59.3	84.2	91.7	72.5	88.6	78.2	76.5
Number of facilities	77	37	79	65	61	61	67	448	9

Table G. Percentage of health facilities with structural tracer items, Senegal SPA 2015

	Non-CEmOC facilities							CEmOC facilities
Domain/Indicator Name	North	Dakar	Thiès	Central	East	South	National Average	National average
**Domain A: Comprehensive emergency obstetric care**								
Parenteral administration of antibiotics	47.5	85.3	55.5	68.3	41.2	57.5	58.9	100.0
Parenteral administration of uterotonic drugs/oxytocin	83.3	98.4	64.3	80.5	87.3	74.3	79.6	100.0
Parenteral administration of anticonvulsants for hypertensive disorders of pregnancy	21.2	44.3	15.1	25.8	34.4	17.1	23.9	100.0
Manual removal of placenta	40.0	41.1	34.0	43.9	39.5	26.4	37.8	100.0
Assisted vaginal delivery	98.3	100.0	93.6	94.3	94.4	99.3	96.4	100.0
Removal of retained products	69.2	78.6	51.9	59.3	66.6	41.0	59.3	100.0
Caesarean section	na	na	na	na	na	na	na	100.0
Blood transfusion	na	na	na	na	na	na	na	100.0
**Domain B: Newborn signal functions and immediate newborn care**								
Neonatal resuscitation	41.3	85.2	55.5	53.9	43.2	43.0	51.2	100.0
Skin-to-skin care	92.0	100.0	100.0	97.9	100.0	100.0	97.7	100.0
Wrap baby	100.0	100.0	100.0	99.2	100.0	100.0	99.8	100.0
Initiate breastfeeding within the first hour	99.3	100.0	100.0	99.2	100.0	99.2	99.5	100.0
**Domain C: General requirements**								
Electricity	42.4	67.1	34.5	41.9	68.7	52.1	47.2	52.9
Improved water source	97.2	100.0	95.4	92.3	86.1	64.0	88.8	100.0
Improved sanitation	93.6	100.0	86.0	84.9	89.6	84.7	88.6	100.0
24/7 skilled birth attendance	2.5	40.0	8.5	10.5	8.4	6.4	10.0	100.0
Emergency transport	45.2	57.1	31.3	49.9	75.1	65.4	51.6	100.0
**Domain D: Equipment**								
Sterilization equipment	35.1	100.0	41.7	37.3	28.3	40.7	42.6	100.0
Delivery bed	98.3	100.0	100.0	96.8	97.9	99.2	98.4	100.0
Examination light	57.1	95.3	60.9	53.2	54.0	66.8	61.3	100.0
Delivery pack	98.4	100.0	100.0	97.2	96.9	100.0	98.6	100.0
Suction apparatus (mucus abstractor)	29.7	73.5	35.3	22.9	9.0	20.4	28.9	100.0
Manual vacuum extractor	3.5	7.9	2.1	2.3	2.3	2.6	3.1	58.8
Vacuum aspirator or D&C kit	35.0	27.9	16.8	33.9	48.6	51.1	35.4	70.6
Partograph	56.8	91.9	62.2	71.5	75.0	57.4	66.4	100.0
Disposable latex gloves	86.9	100.0	84.3	82.0	95.7	91.9	87.9	100.0
Newborn bag and mask	39.1	68.6	42.8	41.8	34.8	53.4	45.1	100.0
Infant scale	87.0	100.0	97.4	88.9	83.8	90.1	90.5	100.0
Blood pressure apparatus (digital or manual)	24.5	93.5	46.9	63.7	86.6	76.4	59.4	100.0
Hand-washing soap and running water or hand disinfectant	88.9	100.0	84.3	87.8	92.7	87.0	88.8	100.0
**Domain E: Medicines and commodities**								
Injectable antibiotic	2.7	64.5	45.9	37.9	34.5	51.5	35.9	88.2
Hydrocortisone available at the facility	41.2	41.8	37.2	44.5	45.5	50.1	43.5	52.9
Injectable uterotonic	30.8	96.9	60.6	57.4	73.9	70.2	59.2	88.2
Skin disinfectant	88.1	93.5	83.2	84.5	97.6	85.3	87.1	100.0
Magnesium sulfate	19.6	54.4	39.1	30.6	35.0	46.4	34.8	100.0
IV solution with infusion set	28.3	56.0	53.0	44.6	41.1	44.8	43.0	100.0
Chlorhexidine for cord cleaning	25.9	59.3	41.8	43.8	85.6	78.7	50.9	58.8
Antibiotic eye ointment for newborn	0.4	62.8	47.4	51.0	69.0	63.4	44.3	41.2

Domain F: Guidelines, staff training and supervision								
Guidelines: Integrated Management of Pregnancy and Childbirth (IMPAC) Guidelines	65.8	56.5	70.4	53.9	59.1	74.5	63.4	47.1
Guidelines: CEmOC Guidelines	52.0	45.0	47.3	25.3	43.0	42.4	40.8	76.5
Guidelines: Guidelines for management of pre-term labor	19.2	14.9	18.8	9.6	9.2	10.2	13.6	47.1
Guidelines on standard precaution	23.5	52.2	38.3	30.4	65.3	54.8	39.4	47.1
Training in neonatal resuscitation	44.6	34.9	35.5	58.3	75.5	65.0	52.5	100.0
Training in early and exclusive breastfeeding	50.7	49.8	35.7	56.3	72.2	63.5	54.0	100.0
Training in newborn infection management (including injectable antibiotics)	28.6	33.3	23.9	50.8	50.9	54.8	41.1	100.0
Training in thermal care	47.8	49.8	33.7	56.8	74.0	66.8	54.0	100.0
Training in cord care	47.8	49.8	33.7	57.0	74.0	65.0	53.7	100.0
Training in IMPAC	18.4	52.9	25.6	34.5	27.0	45.1	32.4	58.8
Training in normal labor and delivery care	42.6	52.9	35.4	48.0	42.4	50.0	45.2	11.8
Training in CEmOC	17.9	26.5	18.6	24.5	17.0	37.0	23.9	11.8
Training in AMTSL	30.9	42.9	26.5	40.8	31.3	45.1	36.6	58.8
Training in KMC	49.0	39.8	30.0	54.8	56.0	61.2	49.7	100.0
Supervision	56.1	37.6	49.1	51.8	35.9	45.7	48.6	88.2
Number of facilities	78	31	56	100	31	65	361	2

Table H. Percentage of health facilities with structural tracer items, Tanzania SPA 2014-15

Domain/Indicator Name	Non-CEmOC facilities: Western	Northern	Central	Southern Highlands	Southern	South West Highlands	Lake	Eastern	Zanzibar	National Average	CEmOC facilities: National average
**Domain A: Comprehensive emergency obstetric care**											
Parenteral administration of antibiotics	43.0	31.9	50.5	20.9	25.2	43.6	28.1	30.4	22.5	33.4	100.0
Parenteral administration of uterotonic drugs/oxytocin	81.9	89.5	90.3	72.1	91.6	95	73.8	85.1	92.3	83.5	100.0
Parenteral administration of anticonvulsants for hypertensive disorders of pregnancy	24.6	18.1	13.0	7.2	3.8	6.8	7.2	22.8	17.5	12.6	100.0
Manual removal of placenta	37.8	31.3	36.5	30.5	59.1	27.4	31.3	27.0	21.0	33.4	100.0
Assisted vaginal delivery	81.6	78.1	78.5	57.2	75.5	55.9	74.0	58.6	29.1	69.3	100.0
Removal of retained products	38.7	21.7	63.8	32.0	33.7	26.3	37.3	25.1	29.3	34.8	100.0
Caesarean section	na	na	na	na	na	na	na	na	na	na	100.0
Blood transfusion	na	na	na	na	na	na	na	na	na	na	100.0
**Domain B: Newborn signal functions and immediate newborn care**											
Neonatal resuscitation	50.1	56.2	59.0	74.4	57.9	61.3	35.4	36.4	35.5	51.7	100.0
Skin-to-skin care	89.7	99.8	91.3	99.4	100.0	95.4	92.4	84.3	81.3	93.6	97.9
Wrap baby	97.5	99.6	99.7	95.7	100.0	96.3	99.7	93.9	96.5	97.9	100.0
Initiate breastfeeding within the first hour	99.7	99.6	92.8	100.0	100.0	100.0	99.3	96.4	88.0	98.4	97.9
**Domain C: General requirements**											
Electricity	87.9	54.2	66.7	63.9	68.4	56.1	72.8	56.6	86.8	66.0	100.0
Improved water source	74.2	69.7	66.2	65.6	61.2	41.8	56.0	65.6	90.3	62.0	88.0
Improved sanitation	25.6	28.1	18.7	25.7	31.7	19.5	45.3	45.7	92.6	32.3	72.1
24/7 skilled birth attendance	18.9	32.7	23.7	18.0	16.9	28.2	39.9	27.0	21.0	27.6	100.0
Emergency transport	57.7	67.5	78.2	42.5	35.8	68.4	74.5	48.4	43.6	61.4	87.5
**Domain D: Equipment**											
Sterilization equipment	28.9	36.7	6.2	12.7	10.1	10.5	15.9	37.0	59.6	20.1	91.3
Delivery bed	100.0	100.0	100.0	98.0	89.0	96.0	99.4	100.0	100.0	98.3	100.0
Examination light	6.5	21.0	6.3	5.6	10.1	26.1	9.0	26.2	35.1	13.9	60.8
Delivery pack	87.9	86.3	89.5	92.2	85.0	95.2	78.8	88.2	52.5	86.8	100.0
Suction apparatus (mucus abstractor)	24.8	25.7	17.7	22.0	22.9	24.3	21.0	21.9	37.4	22.4	73.4
Manual vacuum extractor	2.5	7.0	3.5	1.2	1.0	1.4	7.3	8.3	11.6	4.6	78.8
Vacuum aspirator or D&C kit	11.3	9.5	3.6	4.7	1.3	0.7	10.5	8.4	25.4	7.0	62.0
Partograph	60.1	74.0	43.9	62.0	76.6	51.4	45.9	59.4	59.9	57.1	100.0
Disposable latex gloves	73.4	87.8	95.0	92.7	79.8	83.1	85.3	86.4	96.5	86.1	100.0
Newborn bag and mask	76.4	96.0	85.3	94.1	83.1	76.4	40.5	89.0	45.8	75.9	100.0
Infant scale	82.0	79.5	71.5	74.0	83.9	80.7	76.9	90.2	82.8	79.4	100.0
Blood pressure apparatus (digital or manual)	46.9	77.5	66.9	76.4	57.5	91.4	53.7	65.2	68.6	66.5	95.8
Hand-washing soap and running water or hand disinfectant	46.3	78.4	50.6	87.6	56.8	76.2	56.2	81.2	86.0	67.1	95.8
**Domain E: Medicines and commodities**											
Injectable antibiotic	41.5	35.3	34.8	21.6	35.3	49.9	22.8	26.4	21.0	31.8	70.4
Hydrocortisone available at the facility	25.1	44.6	37.8	26.5	14.8	24.3	21.8	52.4	26.3	31.0	91.7
Injectable uterotonic	71.0	87.6	81.7	84.8	74.0	87.5	71.5	72.7	92.3	78.6	100.0
Skin disinfectant	63.3	70.2	55.6	42.3	71.9	64.9	62.7	60.1	64.0	60.9	85.9
Magnesium sulfate	40.3	41.8	21.1	35.7	50.4	57.0	34.0	48.8	71.2	40.3	97.7

IV solution with infusion set	40.6	55.3	47.4	49.5	28.5	62.5	44.1	45.7	85.5	47.7	95.8
Chlorhexidine for cord cleaning	6.3	19.6	5.0	11.0	9.0	25.4	9.3	9.9	8.2	11.9	20.3
Antibiotic eye ointment for newborn	8.8	34.2	29.3	46.9	9.9	46.3	16.5	30.1	7.0	27.8	51.6
**Domain F: Guidelines, staff training and supervision**											
Guidelines: Integrated Management of Pregnancy and Childbirth (IMPAC) Guidelines	41.2	36.2	16.2	26.7	31.9	21.7	19.7	40.0	32.0	28.0	56.1
Guidelines: CEmOC Guidelines	14.4	5.6	7.5	9.1	3.8	1.5	5.9	22.7	8.2	8.7	26.6
Guidelines: Guidelines for management of pre-term labor	7.7	7.7	8.1	30.4	12.9	4.8	4.4	18.0	7.0	11.2	47.9
Guidelines on standard precaution	12.1	47.2	16.0	38.9	21.9	31.6	19.6	31.0	33.9	27.4	55.0
Training in neonatal resuscitation	79.1	92.1	43.8	76.5	46.2	43.4	21.7	64.5	67.3	55.1	72.9
Training in early and exclusive breastfeeding	74.4	72.6	43.4	69.4	34.6	43.5	23.5	50.1	58.0	49.1	66.5
Training in newborn infection management (including injectable antibiotics)	66.3	54.3	34.8	45.3	30.7	34.6	20.7	34.7	46.3	38.1	54.5
Training in thermal care	76.7	87.4	41.3	61.5	43.0	38.4	22.6	41.3	53.3	48.5	66.5
Training in cord care	76.7	90.5	43.6	65.6	43.0	36.9	21.3	41.6	55.7	49.3	62.3
Training in IMPAC	26.0	24.7	13.2	21.0	10.9	20.0	11.6	13.9	4.7	17.0	24.7
Training in normal labor and delivery care	33.8	28.3	16.1	17.6	24.1	26.5	15.7	16.5	4.7	21.1	37.5
Training in CEmOC	28.6	20.6	9.1	13.7	12.6	15.8	14.1	13.7	2.3	15.6	30.9
Training in AMTSL	33.8	25.0	18.8	17.8	21.3	27.3	16.1	21.3	7.0	21.6	37.4
Training in KMC	59.3	57.8	38.8	55.5	33.8	36.7	19.6	39.8	52.2	40.5	62.2
Supervision	83.0	73.8	81.3	69.2	82.2	86.2	82.7	62.3	89.5	77.6	69.3
Number of facilities	84	109	103	114	65	103	198	113	7	896	8
