# Supplementary material for: Effective coverage of facility delivery in Bangladesh, Haiti, Malawi, Nepal, Senegal, and Tanzania
Source: PLoS One. 2019 Jun 11;14(6):e0217853. doi: 10.1371/journal.pone.0217853 (PMC6559642; doi:10.1371/journal.pone.0217853)
Supplement: S1 File — (PDF) [file pone.0217853.s003.pdf]

The uncertainty of the estimates of effective coverage was assessed with an approximation procedure sometimes referred to as the "delta" method (Hogg and Craig 1965). We refer to the SPA and DHS estimates with the subscripts i=1 and i=2 respectively. The mean readiness score, noted as p_1 for the facilities of a specified type and in a specified region, can be calculated with the coefficient of an OLS regression of readiness scores with no covariates. We call this coefficient b_1 and the standard error of its mean is s_1. The lower and upper ends of the 95% confidence interval for the readiness are $L= b_1$ -1.96* s_1 and $U= b_1$+1.96* s_1. We took into account the effect of survey design in the estimation of standard errors when the SPA was a sample survey. A finite population correction factor was adjusted for in the estimation, given the fact that the SPA sample was drawn from more than 5% of a finite population.

The coverage of facility delivery, noted as p_2, can be estimated using the coefficient b_2 of a logit regression of facility delivery with no covariates. That is, logit(p_2)=log[p_2/(1- p_2)]= b_2. The sampling distribution of b_2 is asymptotically normal with standard deviation s_2. The lower and upper ends of the 95% confidence interval for logit(p_2) are $L= b_2$-1.96* s_2 and $U= b_2$+1.96* s_2. We can calculate the facility delivery coverage as p_2=[exp(b_2)]/[1+exp(b_2)]. If the same anti-logit transformation is applied to L and U, we obtain the lower and upper ends of the confidence interval for coverage. All estimates are adjusted for the survey design.

Effective coverage, p, is defined by $p= p_1 * p_2$. A confidence interval for p is calculated by converting p to the logit scale with

$$\boldsymbol{F} = \boldsymbol{logit}(\boldsymbol{p}) = \log\left(\frac{\boldsymbol{p}}{1-\boldsymbol{p}}\right) = \log\left(\frac{\boldsymbol{p}_1 * \boldsymbol{p}_2}{1-\boldsymbol{p}_1\boldsymbol{p}_2}\right)$$

p_1 and p_2 are functions of the coefficients b_1 and b_2 respectively; the standard errors of b_1 and b_2 are s_1 and s_2 respectively; and the covariance of b_1 and b_2 is 0 because of the independence of the SPA and DHS. Therefore, the sampling variance of F is estimated with the delta method to be

$$\boldsymbol{s}^2 = \left(\frac{\partial \boldsymbol{F}}{\partial \boldsymbol{b}_1}\right)^2 \boldsymbol{s}_1^2 + \left(\frac{\partial \boldsymbol{F}}{\partial \boldsymbol{b}_2}\right)^2 \boldsymbol{s}_2^2$$

and the standard error of F is the square root, s. The partial derivatives in this formula are calculated from the formula for F to be

$$\frac{\partial F}{\partial b_1} = \frac{1}{p_1(1-p_1p_2)} \text{ and } \frac{\partial F}{\partial b_2} = \frac{1-p_1}{1-p_1p_2}$$

We calculate the lower and upper ends of a 95% confidence interval as $L = F$ -1.96* s and $U= F$ +1.96* s, and then apply the anti-logit transformation to L and U to get the lower and upper ends of the confidence interval for $p= p_1 * p_2$ (effective coverage). Similar steps are used to obtain confidence intervals for the aggregated regional and national estimates.
